# LncRNA-PACERR induces pro-tumour macrophages via interacting with miR-671-3p and m6A-reader IGF2BP2 in pancreatic ductal adenocarcinoma

**DOI:** 10.1186/s13045-022-01272-w

**Published:** 2022-05-07

**Authors:** Yihao Liu, Minmin Shi, Xingfeng He, Yizhi Cao, Pengyi Liu, Fanlu Li, Siyi Zou, Chenlei Wen, Qian Zhan, Zhiwei Xu, Jiancheng Wang, Baofa Sun, Baiyong Shen

**Affiliations:** 1 Department of General Surgery, Pancreatic Disease Center, Research Institute of Pancreatic Diseases, Ruijin Hospital, Shanghai Jiaotong University School of Medicine, Shanghai, 200025 China; 2 Research Institute of Pancreatic Diseases, Shanghai Jiaotong University School of Medicine, Shanghai, 200025 China; 3 Department of Zoology, College of Life Science, Nankai University, Tianjin, 300071 China; 4 State Key Laboratory of Oncogenes and Related Genes, Institute of Translational Medicine, Shanghai Jiaotong University, Shanghai, 200025 China; 5 Institute of Translational Medicine, Shanghai Jiaotong University, Shanghai, China

**Keywords:** LncRNA-PACERR, miR-671-3p, KLF12, IGF2BP2, m6A, TAMs, PDAC

## Abstract

**Background:**

LncRNA-PACERR plays critical role in the polarization of tissue-associated macrophages (TAMs). In this study, we found the function and molecular mechanism of PACERR in TAMs to regulate pancreatic ductal adenocarcinoma (PDAC) progression.

**Methods:**

We used qPCR to analyse the expression of PACERR in TAMs and M1-tissue-resident macrophages (M1-NTRMs) which were isolated from 46 PDAC tissues. The function of PACERR on macrophages polarization and PDAC proliferation, migration and invasion were confirmed through in vivo and in vitro assays. The molecular mechanism of PACERR was discussed via fluorescence in situ hybridization (FISH), RNA pull-down, ChIP-qPCR, RIP-qPCR and luciferase assays.

**Results:**

LncRNA-PACERR was high expression in TAMs and associated with poor prognosis in PDAC patients. Our finding validated that LncRNA-PACERR increased the number of M2-polarized cells and facilized cell proliferation, invasion and migration in vitro and in vivo. Mechanistically, LncRNA-PACERR activate KLF12/p-AKT/c-myc pathway by binding to miR-671-3p. And LncRNA-PACERR which bound to IGF2BP2 acts as an m6A-dependent manner to enhance the stability of KLF12 and c-myc in cytoplasm. In addition, the promoter of LncRNA-PACERR was a target of KLF12 and LncRNA-PACERR recruited EP300 to increase the acetylation of histone by interacting with KLF12 in nucleus.

**Conclusions:**

This study found that LncRNA-PACERR functions as key regulator of TAMs in PDAC microenvironment and revealed the novel mechanisms in cytoplasm and in nucleus.

**Supplementary Information:**

The online version contains supplementary material available at 10.1186/s13045-022-01272-w.

## Background

Pancreatic ductal adenocarcinoma (PDAC), a major type of pancreatic cancer, is the most aggressive and lethal alimentary tract malignancies, lacking clinical features, with extremely grave prognosis and a dismal five-year survival rate of 10% [1, 2]. At present, the main treatments for PDAC patients were surgery and chemotherapy. However, many patients who were diagnosed with PDAC at advanced stage lost the opportunity of curative resection and gemcitabine-based comprehensive treatments have only provided minimal survival benefits [3]. Up to now, although scientists got the groundbreaking achievements of immunotherapy in the past few years, immunotherapy treatment have also failed to yield desired efficacy in PDAC [4, 5]. Thus, novel therapeutic approaches are desperately required for PDAC patients.

The grave prognosis of PDAC is associated with an extensive fibrous inflammatory TME with a large infiltration of cancer-associated fibroblasts (CAFs) and immune cells which are hijacked by cancer cells to provide immunosuppressive microenvironments for the growth of PDAC [6]. The TME in PDAC consists of pancreatic cancer cells, extracellular matrix (ECM) and stromal cells which are mainly composed of pancreatic stellate cells (PSCs), regulatory T cells, myeloid-derived suppressor cells (MDSCs) and TAMs [7]. TAMs, as the main immune cells, constitute 11% of all cellular composition in the PDAC TME [8]. Macrophages are highly dynamic cells, capable of acquiring phenotypes (M1 and M2) and functions in response to environmental stimuli [9, 10]. The vast majority of TAMs in the TME are differentiated into the M2 phenotype that promotes tumour growth, metastasis [11, 12]. Extensive studies have found that the high degree of TAMs infiltration is corelated with poor prognosis of PDAC [13, 14]. Therefore, targeting TAMs is an important direction for immunotherapy of PDAC.

In recent years, long non-coding RNAs (lncRNAs) have attracted much scientific attention due to their high abundance but limited protein-coding capacity. Numerous studies have demonstrated that lncRNAs are involved in many biological processes of tumour, including immune response, proliferation and metastasis of tumour cells [15, 16]. In addition, most studies have identified mechanisms of lncRNAs including RNA stability, protein function and transcriptional regulation [17, 18]. Many lncRNAs have been shown to play key regulatory roles in the TME of pancreatic cancer, but their molecular functions in TAMs remain unclear. Therefore, it is crucial to elucidate the molecular mechanisms of lncRNAs in TAMs. The mechanisms of lncRNAs are determined by their subcellular localization. On the one hand, lncRNAs in the cytoplasm can function as competitive endogenous RNAs (ceRNAs), alleviating the repressive effect of miRNAs on mRNA expression by sequestering miRNAs from spongy target mRNAs [19], and on the other hand, lncRNAs can bind RNA-binding proteins [20]. The lncRNAs in the nucleus can be involved in the transcriptional regulation of genes [21].

LncRNA-PACERR has been widely reported as an activator of pro-tumour macrophages and plays a role in promoting proliferation, migration and invasion of tumour cells in a variety of cancers including colorectal cancer and osteosarcoma [22, 23]. However, the biological functions and molecular mechanisms of LncRNA-PACERR in pancreatic cancer have been rarely reported. miR-671-3p has been reported as an oncogene that inhibits the progression of cancers [24]. KLF12 is a transcription factor with a zinc finger structure that has been extensively studied and plays a key role in cell proliferation and invasion [25, 26]. In recent years, it has been shown that KLF12 can promote tumour proliferation and invasion through PI3K/AKT/c-myc axis [27]. N6-methyladenosine (m6A), the most abundantly modified form of RNAs in eukaryotes, regulates gene expression and cell fate [28]. Such modifications are subject to the involvement of m6A regulators, including 'writer', 'eraser' and 'reader' proteins. For example, insulin-like growth factor 2 mRNA-binding protein 2 (IGF2BP2) is a member of the m6A reader family, which functions primarily by recruiting cofactor proteins to stabilize m6A-modified transcripts [29]. Some studies have reported that c-myc is one of the targets of IGF2BP2 [30].

In this study, we provided the expression levels of LncRNA-PACERR in TAMs in human pancreatic cancer, validated the pro-oncogenic functions of LncRNA-PACERR through biological experiments and revealed that LncRNA-PACERR sponges miR-671-3p to activate the KLF12/AKT/c-myc pathway and interacts with IGF2BP2 to enhance the stability of KLF12 and c-myc. In the meantime, KLF12 promotes the transcription of LncRNA-PACERR in the nucleus by forming KLF12/LncRNA-PACERR complex to recruit histone acetyltransferase-EP300.

## Methods

### Clinical samples

Fresh PDAC tumour tissues and normal tissues were collected from patients in Ruijin Hospital of Shanghai Jiaotong University School of Medicine from 2019 to 2021. Tissue microarrays (TMAs) from 110 PDAC patients were produced in 2021. After tumour and normal tissues were excised from PDAC patients, total single cells were labelled with Anti-human CD163-phycoerythrin (PE) and Anti-human CD80b-phycoerythrin (PE) monoclonal antibodies, and then TAMs and M1-NTRMs combined with Anti-PE microbeads and were isolated by magnetic-activated cell sorting (MACS). The study was approved by the Research Ethics Committee. All patients signed informed consent forms. The information of paired tissues from 46 PDAC patients and TMAs from 110 PDAC patients is listed in Additional file 1: Table S1 and Additional file 2: Table S2.

### Cell culture and reagents

THP-1 and PATU-8988 were cultured in RPMI-1640 which contained 10% foetal bovine serum (FBS) (LONSERA, Shanghai Shuangru Biology Science & Technology Co.,Ltd.) and streptomycin. PANC-1, 0037 and HEK-293 T cells were cultured in DMEM which was added 10% FBS and streptomycin. 100 ng/ml phorbol 12-myristate 13-acetate (PMA) were used to convert THP-1 into macrophages for forty-eight hours. THP-1 (treated with PMA for 48 h) co-cultured with pancreatic cancer cells for 48 h was considered as THP-1-derived TAMs.

### Plasmids and stable cell lines

It was as previously described to get stable cell lines [31]. IGF2BP2, METTL14 and KLF12 knockdown plasmids, miR-671-3p mimics plasmids, miR-671-3p inhibitor plasmids and overexpression of PACERR, IGF2BP2, KLF12 plasmids were purchased from BioeGene Co., Ltd. LncRNA-PACERR knockdown lentivirus, KLF12-Flag and KLF12-Mut-Flag overexpressed plasmids were gotten from GeneChem, and the related sequences of plasmids are listed in Additional file 3: Table S3. Lentivirus (MOI: 100) with 5 μg/ml polybrene was used to establish stable cell lines.

### Quantitative real-time PCR analysis

 RNA Express Total RNA Kit M050 (New Cell & Molecular Biotech) were used to extract RNA from TAMs, M1-NTRMs and THP-1 cells. PARIS Kits (Invitrogen) were applied to separate RNA fractions of THP-1 cells from nucleus and cytoplasm. cDNA was synthesized by using Evo M-MLV RT Kit with gDNA Clean for qPCR II AG11711 (Accurate Biotechnology(Human)Co.,Ltd.). SYBR Green Premix Pro Taq HS qPCR Kits AG11721 (Accurate Biotechnology(Human)Co.,Ltd.) was used for quantitative real-time PCR assays. GAPDH Total RNA from TAMs, monocytes from patients’ peripheral blood and cell lines used in this study was extracted in TRIzol (Invitrogen, USA). RNA in nucleus or cytoplasm was extracted with a PARIS Kit (Invitrogen, USA). We reverse transcribed synthesized cDNA by using HiScript III RT SuperMix for qPCR (+ gDNA wiper) (Vazyme). GAPDH was designated as a positive control of internal mRNA and PACERR. U6 was used as positive control of internal miRNA and PACERR which was in nucleus. The related primers are given in Additional file 4: Table S4.

### Western blot

Western blot assay was as described previously [32]. PAGE Gel Fast Preparation Kit was used for WB assay. (Epizyme Biotech, PG112) The protein expression was detected by using antibodies of KLF12, c-myc, PI3K, p-AKT, PTEN, IGF2BP2, EP300, Mettl3, Mettl14, WTAP, NMT1, FLAG and GAPDH. Details of antibodies are listed in Additional file 5: Table S5. The expression of GAPDH was used as internal reference.

### Dual luciferase reporter assay

Dual luciferase reporter assay was as described previously [33]. HEK-293 T cells and THP-1 cells were cultured in 6-well plates at 2 × 10^5^ cells per well for twenty-four hours. HEK-293 T were co-transfected with LncRNA-PACERR and wild-type miR-671-3p reporter plasmids (Biogene). THP-1 cells were transfected reporter plasmids of KLF12 and promoter sequences of LncRNA-PACERR. Dual luciferase reporter assay kit DL101-01(Vazyme Biotech Co.,Ltd) was used to measure luciferase activity.

### In vivo assays

5 × 10^6^ stable LncRNA-PACERR knockdown (KD) and control THP-1 cell lines treated with PMA and 1 × 10^7^ PATU-8988/0037 wild-type cells were co-injected subcutaneously into per BALB/c nude mice. After four weeks, we measured the width, length and weight of tumour. Width x width x length/2 was used as formula to calculate tumour volume. For liver metastases model, 5 × 10^6^ stable LncRNA-PACERR knockdown and control THP-1 cells treated with PMA for two days and 1 × 10^7^ PATU-8988/0037 wild-type cells were co-injected into spleens of per BALB/c nude mice. After 4–6 weeks, we obtained liver tissues after dissecting mice.

### RIP-qPCR assay

RIP assay was as described previously [34]. MagnaRIP RNA-Binding Protein Immunoprecipitation Kit (Millipore) was applied for this experiment. The details of IGF2BP2, m6A, FLAG, KLF12 and IgG antibodies arelisted in Additional file 5: Table S5.

### ChIP-qPCR assay

It was as described previously for ChIP assay [35]. KLF12, EP300, H3K27ac, H3K27me3 and IgG were used for ChIP. The information of antibodies is given in Additional file 5: Table S5, and related primers are given in Additional file 4: Table S4.

### Statistical analysis

Linux, R platform, GraphPad Prism 8 and Zesis were applied for analysis. And the R platform was used for statistical analysis. Three to five repeated experiments were performed in related figures. Data are presented as the mean ± SD, and the difference between the two groups was calculated by the paired two-tailed Student's t-test, one-way ANOVA and Chi-square test.

Additional methods can be found in Additional file 7: Supplementary Methods.

## Results

### LncRNA-PACERR is overexpressed in TAMs and elevated infiltration of PACERR^+^ TAMs correlates with poor prognosis of patients with PDAC

**Fig. 1:**
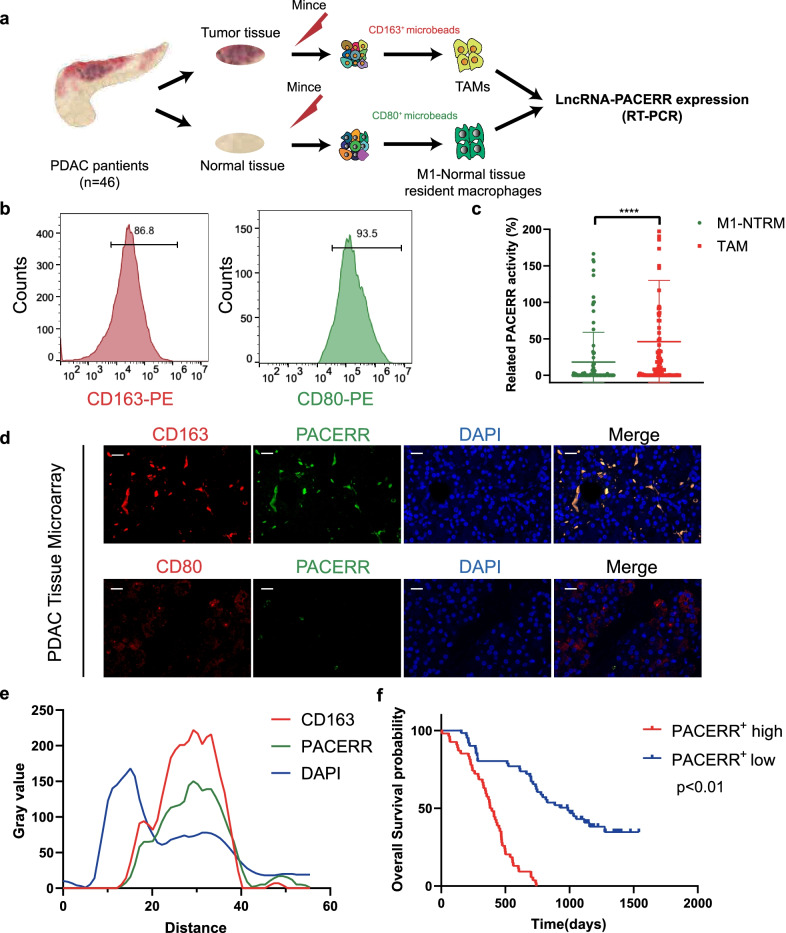
LncRNA-PACERR expression in TAMs is activated in PDAC tissues and is associated with poor prognosis. **A** Schematic illustration of qRT-PCR forty-six sample preparation (*n* = 46). Tumour-associated macrophages (TAMs) from forty-six PDAC tissue samples were enriched with CD163 positive selection. M1 macrophages in normal tissue-resident macrophages (M1-NTRMs) from PDAC adjacent normal tissue samples of the same patient were enriched with CD80 positive selection. **B** Purity of CD163^+^ cells and CD80^+^ by CD163 and CD80 microbeads sorting. **C** Expression of LncRNA-PACERR in 46 pairs of TAMs and M1-NTRMs from PDAC. *P < 0.05; **P < 0.01; ***P < 0.001; ****P < 0.0001. **D** Colocalization of LncRNA-PACERR (green) and CD163/CD80 (red) in 110 clinical samples of pancreatic ductal adenocarcinoma (PDAC) as shown by fluorescence microscopy. DAPI staining (blue) shows the nuclei (DNA). Scar bar: 50 μm. **E** Staining intensity of LncRNA-PACERR, CD163 and DAPI on the immunofluorescence from TMAs of 110 PDAC patients. Green represents LncRNA-PACERR. Red represents CD163. Blue represents DAPI. **F** Kaplan–Meier survival curve presenting the overall survival of 110 PDAC patients, grouped according to the extent of LncRNA-PACERR^+^ TAMs infiltration

To characterize the expression level of LncRNA-PACERR in TAMs in PDAC, we isolated CD163^+^ cells which represented TAMs from tumour core of PDAC with a volume of 0.5 cm^3^ and CD80^+^ cells which were regarded as M1 macrophages in normal tissue-resident macrophages (M1-NTRMs) from para-cancerous tissues with a volume of 0.5 cm^3^ from 46 PDAC patients by using magnetic-activated cell sorting (MACS) (Fig. 1a). Meanwhile, we used immunohistochemistry to present the expression of CD163 and CD80 and confirmed that PDAC patients with high infiltration of CD163 + TAMs had a worse prognosis (Additional file 6: Fig. S1a, b). We ensured high purity of sorted cells by flow cytometric analysis (Fig. 1b). qPCR analysis of 46 paired TAMs and M1-NTRMs in PDAC and FISH-immunofluorescence analysis of PDAC tissue microarrays (TMAs) which were composed of tumour tissues and non-tumour tissues from 110 patients with PDAC demonstrated that LncRNA-PACERR is significantly more highly expressed in TAMs compared to M1-NTRMs (Fig. 1c, d and Additional file 1: Table S1). And there was a strong relationship of co-localization between CD163 and LncRNA-PACERR (Fig. 1e). To further inspect the clinical prognostic value of LncRNA-PACERR^+^ TAMs, we counted the positive rate of LncRNA-PACERR in TAMs from TMAs and divided 110 pancreatic cancer patients into PACERR high expression group and PACERR low expression group based on the median of the positive rate. Kaplan–Meier analysis showed that high infiltration of LncRNA-PACERR^+^ TAMs was associated with a poor prognosis in PDAC (*P* < 0.01) (Fig. 1f). Collectively, these results revealed that more LncRNA-PACERR^+^ TAMs were associated with a worse prognosis of PDAC.

### LncRNA-PACERR^+^ TAMs are characterized by M2-polarized macrophages and promote proliferation, migration and invasion of pancreatic cancer cells in vitro and in vivo

**Fig. 2:**
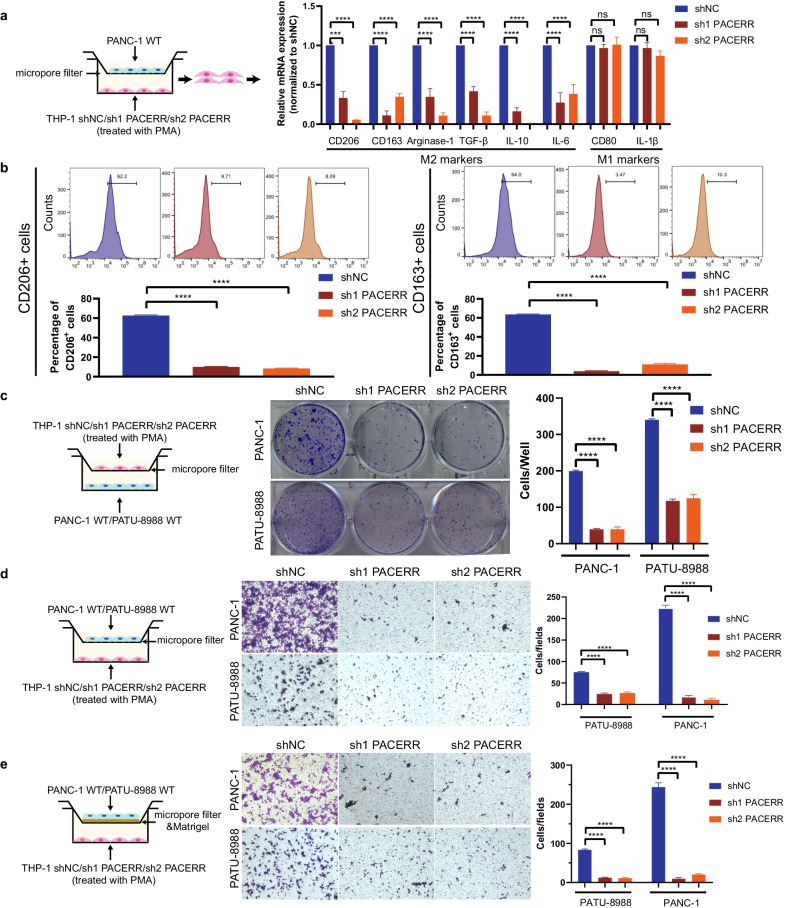
Knockdown of LncRNA-PACERR hinders the M2 polarization and pro-tumour functions of THP-1-derived TAMs in vitro*.* **A** qPCR analysis of the relative expression of M2 markers (Arginase-1, CD163, TGFβ, CD206, IL-10 and IL-6) and M1 marker (CD80, IL-1β) in THP-1-derived TAMs after LncRNA-PACERR knockdown. THP-1 cells were treated with PMA and co-cultured with PANC-1 cells for two days. Data are shown as the results from three independent experiments. **B** Flow cytometric analysis of the expression of M2 markers (CD163 and CD206) in THP-1-derived TAMs after LncRNA-PACERR knockdown. THP-1 cells were treated with PMA and co-cultured with PANC-1 cells for two days. Data are shown as the results from two independent experiments. **C**–**E** Proliferation (**C**), migration (**D**) and invasion (**E**) capacity of PATU-8988 or PANC-1 cells co-cultured with THP-1-derived TAMs (shNC/ shPACERR). shNC means that cells were transfected in negative control plasmids. *P < 0.05; **P < 0.01; ***P < 0.001; ****P < 0.0001

**Fig. 3:**
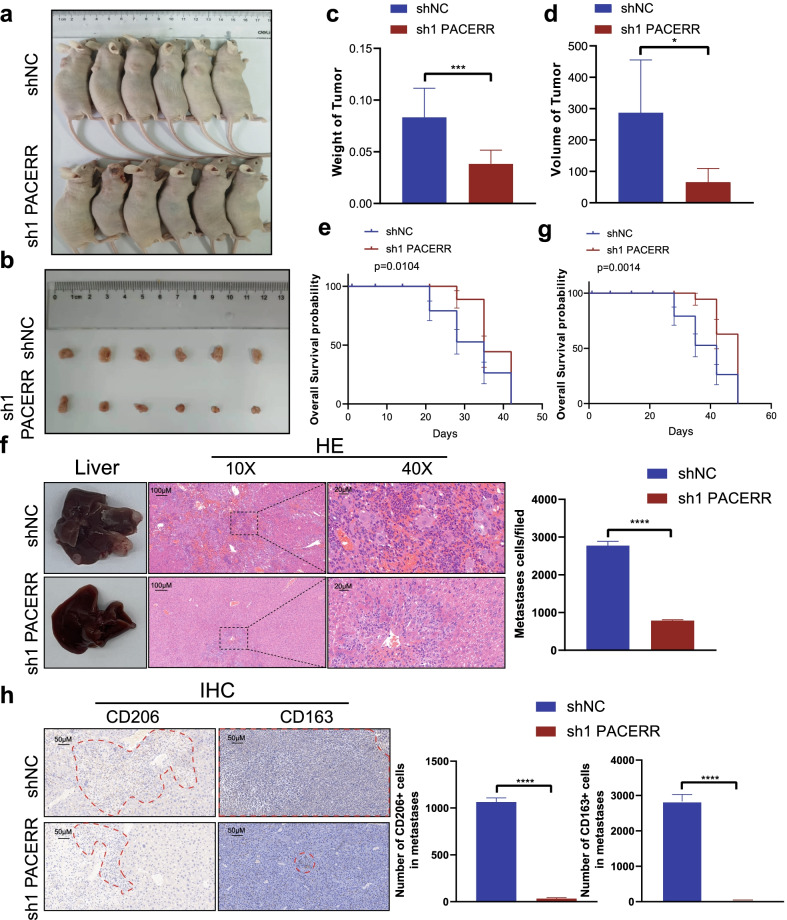
LncRNA-PACERR^+^ TAMs facilitate PDAC cell growth and liver metastasis in vivo. **A** Images of BALB/c nude mice which were co-injected with THP-1 cells and PATU-8988 cells subcutaneously. **B** Images of subcutaneous tumours. **C**, **D** Tumour weights and volumes of the subcutaneous xenografts. **E**, **G** Kaplan–Meier survival curve presenting the overall survival of BALB/c nude mice (shNC/sh1 PACERR groups) (*n* = 6). **F** Representative images of liver metastasis and the number of metastatic cells in PDAC mouse model, in which PATU-8988 cells mixed with TAMs (THP-1 shNC/sh1 LncRNA-PACERR) were injected into the spleens of BALB/c nude mice. Data iare shown as the results from three independent experiments. **H** Representative images of IHC (CD163 and CD206) of liver metastasis and the number of CD163^+^ and CD206^+^ cells in metastatic foci from liver tissues. *P < 0.05; **P < 0.01; ***P < 0.001; ****P < 0.0001

To clarify the function of PACERR, we used THP-1 cell lines and PATU-8988/PANC-1 cells to construct a co-culture model of TAMs in vitro (Fig. 2a). qPCR analysis confirmed that the expression levels of M2 macrophages markers CD206, CD163, Arginase-1, TGFβ, IL-10 and IL-6 were downregulated after knockdown of LncRNA-PACERR in TAMs (Fig. 2a, Additional file 6: Fig. S2a). Flow cytometry revealed that depleted expression of LncRNA-PACERR in THP-1 derived TAMs resulted in decreased ratio of CD206^+^ and CD163^+^ macrophages (Fig. 2b). As many previous studies have reported that TAMs promote tumour cell proliferation, migration and invasion [36], we explored the pro-tumour function of TAMs through a co-culture model of pancreatic cancer cells and macrophages in vitro. Firstly, we combined previous studies [37] and demonstrated that THP1 could exert toxicity to pancreatic cancer cells, which will be the negative control for each of the following tests (Additional file 6: Fig. S2b-d). As expected, we found that knockdown of LncRNA-PACERR in TAMs inhibits pancreatic cancer cell proliferation based on colony formation and CCK8 assays (Fig. 2c and Additional file 6: Fig. S2e, f). And we investigated that LncRNA-PACERR in TAMs increased the migration and invasion ability of pancreatic cancer cells by using Transwell assays (Fig. 2d, e). To further validate the impact of LncRNA-PACERR in TAMs, PATU-8988 cells mixed with LncRNA-PACERR knockdown or negative control macrophages (THP-1 shNC/sh-LncRNA-PACERR cells) were injected into subcutaneous of BALB/c nude mice or into the spleens of BALB/c nude mice (*n* = 6 per group). We found that subcutaneous volumes and weights of tumour in the knockdown of LncRNA-PACERR group were decreased in the knockdown of LncRNA-PACERR group. Then, we used another 2 groups to show the prognosis of mice and observed that mice in the knockdown of LncRNA-PACERR group had longer overall survival compared to those in the negative control group (Fig. 3a–e). To verify the ability of LncRNA-PACERR to promote tumour growth, IHC staining of subcutaneous tumour tissues was performed with Ki-67 antibody. The expression of Ki-67 was downregulated in the LncRNA-PACERR knockdown group (Additional file 6: Fig. S3a). For metastasis mouse models, we observed that these mice had similar prognosis as the mice in the subcutaneous tumour model and the number of metastatic cells in liver was diminished and the number of CD163^+^ cells and CD206^+^ cells in metastatic foci (number of CD163 + /CD206 + cells per field) was decreased after knockdown of LncRNA-PACERR in TAMs (Fig. 3f–h). In addition, we obtained similar findings by using primary pancreatic cancer cells (0037) from a PDAC patient to construct subcutaneous tumour mouse model and liver metastasis mouse model, respectively (Additional file 6: Fig. S3b-f) [38].

### LncRNA-PACERR upregulated the KLF12/p-AKT/c-myc axis by sponging miR-671-3p

**Fig. 4:**
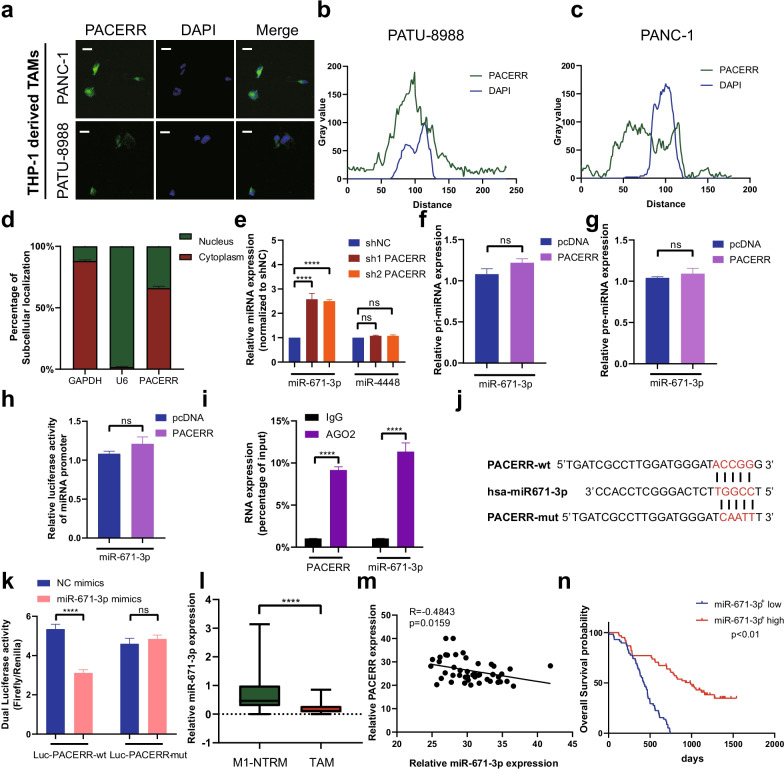
LncRNA-PACERR functions as a ceRNA to sponge miR-671-3p in TAMs. **A** Fluorescence in situ hybridization (FISH) of LncRNA-PACERR (green) in THP-1-derived TAMs (co-cultured with PATU-8988 or PANC-1). DAPI staining (blue) shows the nuclei. Scar bar: 10 μm. **B**, **C** Grey value of LncRNA-PACERR and DAPI on the FISH from THP-1 derived TAMs. Green represents LncRNA-PACERR. Blue represents DAPI. **D** Expression levels of LncRNA-PACERR in the cytoplasm and nucleus in THP-1-derived TAMs. **E** Expression of two potential target miRNAs (miR-671-3p and miR-4448) in THP-1 derived TAMs after LncRNA-PACERR knocked down. **F**, **G** Expression of pri-miR-671-3p (**F**) and pre-miR-671-3p (**G**) in THP-1 derived TAMs transfected with empty control or pcDNA-LncRNA-PACERR. **H** Promoter luciferase activity of miR-671-3p in 293-T cells overexpressing LncRNA-PACERR. **I** RIP assay was performed using rabbit AGO2 and IgG antibodies in THP-1 derived TAMs. Relative expression levels of LncRNA-PACERR and miR-671-3p were determined by qRT-PCR. **J**, **K** Dual luciferase activity in 293-T cells co-transfected with LncRNA-PACERR wild-type or mutant sequence and miR-671-3p mimics. **L** Expression of miR-671-3p in 46 pairs of TAMs and M1-NTRMs from PDAC patients. **M** Correlation analysis between LncRNA-PACERR and miR-671-3p using expression data from 46 pairs of TAMs. **N** Kaplan–Meier survival curve presenting the overall survival of 110 PDAC patients, grouped according to the extent of miR-671-3p^+^ TAMs infiltration

**Fig. 5:**
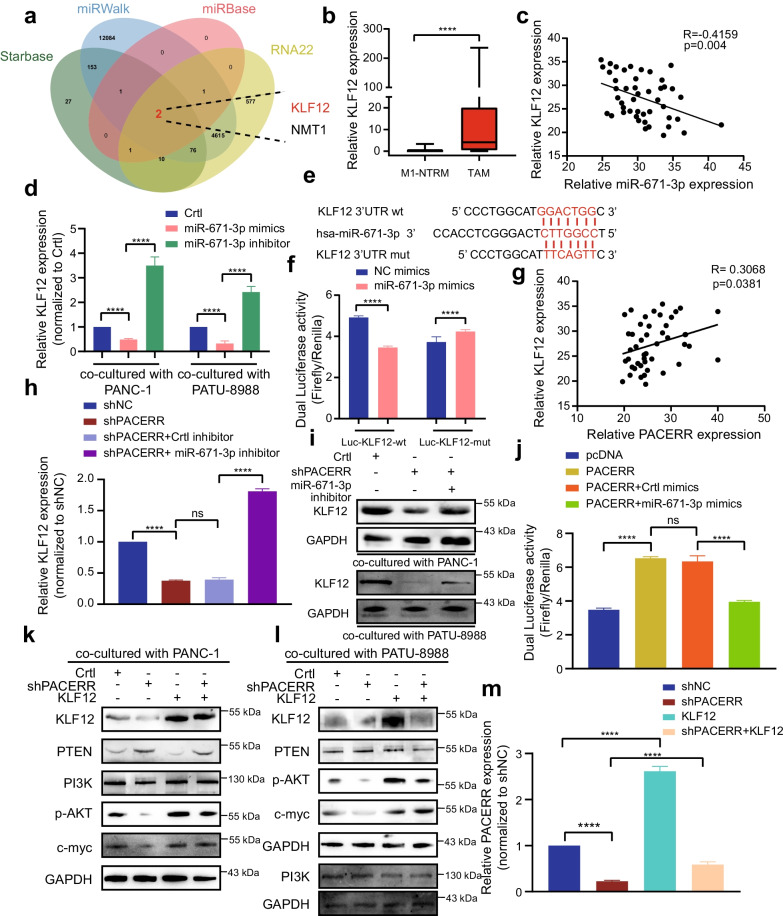
MiR-671-3p directly binds to the 3′UTR of KLF12 and regulates the KLF12/AKT/c-myc axis. **A** Predicted target genes of miR-671-3p using Starbase, miRWalk, miRbase, RNA22 and the two overlapping target genes (NMT-1 and KLF12). **B** Expression of KLF12 in 46 pairs of TAMs and M1-NTRMs from PDAC patients. **C** Correlation analysis between miR-671-3p and KLF12 using data from 46 pairs of PDAC patients. **D** mRNA expression of KLF12 in THP-1 derived TAMs (co-cultured with PATU-8988 or PANC-1 cells) transfected with empty control or miR-671-3p mimics or inhibitor. **E**, **F** Dual luciferase activity in HEK-293 T cells co-transfected with the KLF12 wild-type or mutant sequence (**E**) and miR-671-3p mimics. **G** Correlation analysis between LncRNA-PACERR and KLF12 in TAMs from 46 PDAC patients. **H** mRNA expression of KLF12 in THP-1 derived TAMs co-transfected with the LncRNA-PACERR knocked down vector and miR-671-3p inhibitor. **I** Protein levels of KLF12 in THP-1 derived TAMs (co-cultured with PATU-8988 or PANC-1 cells). **J** Dual luciferase activity in HEK-293 T cells co-transfected with LncRNA-PACERR overexpression vector and miR-671-3p mimics. **K**, **L** Potein expression of PI3K/KLF12/AKT/c-myc axis in THP-1 derived TAMs (co-cultured with PANC-1 (**K**) or PATU-8988 cells (**L**)). **M** LncRNA-PACERR expression was evaluated by qRT-PCR in THP-1 derived TAMs with indicated treatment. The data are presented as the mean ± SD of three independent experiments. *P < 0.05; **P < 0.01; ***P < 0.001; ****P < 0.0001

To unravel the molecular mechanisms by which LncRNA-PACERR exerts its function in promoting malignant tumour progression, we identified the subcellular location of LncRNA-PACERR which revealed the main distribution of LncRNA-PACERR in the cytoplasm and nucleus in TAMs by using FISH and cellular fractionation assays (Fig. 4a–d and Additional file 6: Fig. S4a). To explore the mechanism of LncRNA-PACERR in the cytoplasm, we thought of the ceRNA mechanism as one of the most common mechanisms for LncRNAs in the cytoplasm [39, 40]. Hence, we were curious whether LncRNA-PACERR could play the role of ceRNA to bind with miRNAs. We examined the RNA expression of two potential LncRNA-PACERR target miRNAs (miR-4448 and miR-671-3p) predicted by miRTarBase websites after LncRNA-PACERR knockdown using qPCR (Fig. 4e). MiR-671-3p was upregulated and miR-4448 was no changed after knockdown of LncRNA-PACERR in THP-1 derived TAMs (Fig. 4e). Therefore, we selected miR-671-3p for further study. To clarify whether LncRNA-PACERR is involved in miRNA transcriptional regulation through pri-miRNA or pre-miRNA synthesis, we demonstrated that LncRNA-PACERR has no significant effect on the expression of pri-miRNA or pre-miRNA or promoter activity (Fig. 4f–h). In addition, RIP results showed that AGO2 bound significantly to both LncRNA-PACERR and miR-671-3p (Fig. 4i). To further clarify whether miR-671-3p could directly bind to LncRNA-PACERR, we designed mutant plasmids based on predicted binding sites and determined the interaction of miR-671-3p with LncRNA-PACERR by dual luciferase reporter assays (Fig. 4j, k and Additional file 6: Fig. S4b). In order to better demonstrate the binding between LncRNA-PACERR and miR-671-3p, we used RNA pull-down assays to verify the binding exactly (Additional file 6: Fig. S4c). Moreover, we used 46 paired clinical samples to detect expression of LncRNA-PACERR and miR-671-3p and found that miR-671-3p was lower expressed in TAMs than those in M1-NTRMs and was negatively correlated with LncRNA-PACERR (Fig. 4i, m). To assess the clinical prognosis of miR-671-3p, 110 patients who were made up the PDAC TMAs were divided into two groups (miR-671-3p^+^ high group and miR-671-3p^+^ low group) based on the median of density of miR-671-3p^+^ TAMs infiltration according to RNA FISH and IF (Fig. 4n). Kaplan–Meier analysis of overall survival from 110 patients revealed that higher miR-671-3p expression was associated with better prognosis of PDAC (Fig. 4n). To clarify whether miR-671-3p is involved in the pro-tumour effects of LncRNA-PACERR in pancreatic cells, we transfected a miR-671-3p inhibitor plasmid into THP-1 cells in which LncRNA-PACERR was knocked down and found that miR-671-3p downregulation could increase the expression of M2 macrophages markers via qPCR and flow cytometry (Additional file 6: Fig. S4d-f). Based on results of CCK8 and colony formation assays, the depletion of miR-671-3p upregulated PATU-8988 and PANC-1 cell proliferation (Additional file 6: Fig. S5a-c). Transwell assay results demonstrated that PATU-8988 or PANC-1 cells co-cultured with THP-1 cells in miR-671-3p inhibitor group rescued the ability of migration and invasion (Additional file 6: Fig. S5d-e). Then, we clarified that miR-671-3p could inhibit pancreatic cancer cell proliferation, invasion and migration in TAMs (Additional file 6: Fig. S6a-c) Subsequently, we referenced two potential target genes from four online prediction websites (Starbase, miRWalk, miRbase and RNA22) for miR-671-3p and observed that the expression of NMT1 was not significantly changed after knockdown of LncRNA-PACERR (Additional file 6: Fig. S7a). Finally, we identified the most famous target gene-KLF12 which has been reported in PDAC (Fig. 5a). qPCR analysis of 46 paired PDAC samples and IF (CD163 and KLF12) analysis of TMAs (*n* = 110) validated that KLF12 was high expressed in TAMs and was associated with a poor prognosis (Fig. 5b and Additional file 6: Fig. S7b, c). To explore whether miR-671-3p could directly sponge KLF12, we first found a negative correlation between KLF12 and miR-671-3p by measuring their RNA expression levels in 46 paired PDAC samples (*P* = 0.004) (Fig. 5c). In addition, KLF12 mRNA expression levels decreased after transfection with miR-671-3p mimics and increased after transfection with miR-671-3p inhibitors in THP-1 derived TAMs (Fig. 5d). Dual luciferase reporter assays revealed that there were diminish of luciferase activity in the Luc-KLF12-wt group and no change of luciferase activity in the Luc-KLF12-mut group after HEK-293 T cells were transfected with miR-671-3p mimics (Fig. 5e, f). Additionally, we found that the expression of KLF12 were positively correlated with the expression levels of LncRNA-PACERR in TAMs (Fig. 5g and Additional file 6: Fig. S7d). In THP-1 derived TAMs, low expression levels of LncRNA-PACERR significantly downregulated KLF12 mRNA and protein expression. Transfection of miR-671-3p inhibitors rescued KLF12 expression in THP-1 with LncRNA-PACERR knockdown (Fig. 5h, i). Meanwhile, transfection of the LncRNA-PACERR overexpression plasmid increased Luc-KLF12-wt luciferase activity, while simultaneous transfection of LncRNA-PACERR and miR-671-3p mimics attenuated this effect in HEK-293 T (Fig. 5j). It has been reported that KLF12 promotes malignant tumour progression via the AKT/c-myc pathway [27, 41]. As expected, western blot analysis showed that KLF12 regulated by LncRNA-PACERR significantly promoted AKT phosphorylation which increased c-myc expression through downregulation of PTEN in THP-1 derived TAMs (Fig. 5k, l). Furthermore, after CD163 + TAMs were sorted from tumour tissues from subcutaneous mice, we observed that the expression of KLF12 and c-myc was significantly decreased in the LncRNA-PACERR knockdown group (Additional file 6: Fig. S7e). To further validate that KLF12 is participated in the pro-tumour effects of LncRNA-PACERR in TAMs, we got similar results to LncRNA-PACERR by using qPCR, flow cytometry, CCK8, colony formation and Transwell assays, and found that overexpression of KLF12 can promote the expression of LncRNA-PACERR (Fig. 5m, Additional file 6: Fig. S7f, g and Fig S8a-e). Next, we found that KLF12^+^ TAMs could enhance the ability of pancreatic cancer cell proliferation, invasion and migration (Additional file 6: Fig. S9a-c). Together, these results indicated that LncRNA-PACERR could release KLF12 by sequestering miR-671-3p, thereby achieving TAMs to facilitate malignant progression of pancreatic cancer via the KLF12/AKT/c-myc pathway.

### KLF12/LncRNA-PACERR complex recruits EP300 to facilitate transcription of LncRNA-PACERR in nucleus

**Fig. 6:**
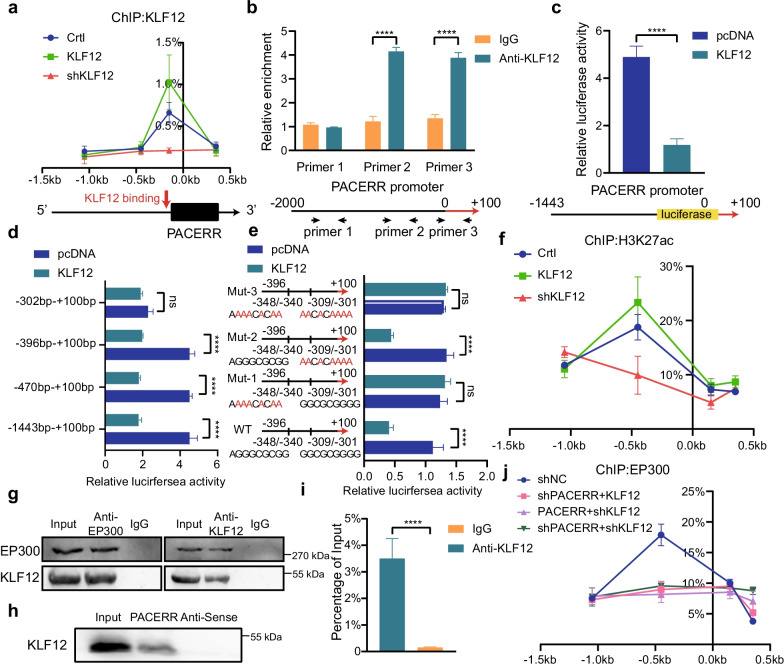
KLF12 binds directly to LncRNA-PACERR and recruits EP300 to the promoter region of LncRNA-PACERR in a LncRNA-PACERR-dependent manner. **A** Association of KLF12 with the promoter region of LncRNA-PACERR in THP-1-derived TAMs (Crtl/KLF12 OE / shKLF12) analysed by ChIP-qPCR. **B** ChIP assays showed endogenous KLF12 binding to the LncRNA-PACERR gene promoter. **C**, **D** HEK293T cells were co-transfected with LncRNA-PACERR promoter–luciferase truncations and KLF12 plasmids, and the luciferase activity was determined using a dual luciferase reporter assay after 48 h. **E** Dual luciferase assay of HEK293T cells co-transfected with firefly luciferase constructs containing the wild-type or mutant KLF12 potential binding sites of LncRNA-PACERR promoter and KLF12 plasmids were performed. **F** Association of H3K27ac with the promoter region of LncRNA-PACERR in THP-1-derived TAMs (Crtl/ KLF12 OE/ shKLF12) analysed by ChIP-qPCR. **G** Results of coimmunoprecipitation (Co-IP) in THP-1-derived TAMs. Normal rabbit IgG was used as a negative control. **H** WB validation of KLF12 proteins pulled down with biotin-labelled LncRNA-PACERR is shown. **I** RNA immunoprecipitation (RIP) was performed using a KLF12-specific antibody. Eluted KLF12-binding RNAs were reverse transcribed, and qPCR was performed with primers specific for LncRNA-PACERR. Normal rabbit IgG (IgG) was used as a negative control. Data are shown as the results from three independent experiments. **J** Association of EP300 with the promoter region of LncRNA-PACERR analysed by ChIP-qPCR in THP-1-derived TAMs (shNC/ shLncRNA-PACERR + KLF12 OE/shKLF12 + LncRNA-PACERR OE/ shKLF12 + shLncRNA-PACERR)

Some studies have reported that KLF12 enhances tumour malignant progression by participating in transcriptional regulation of oncogenes [42]. Therefore, we speculate that KLF12 may promote the transcription of LncRNA-PACERR in the nucleus based on Fig. 5m. We determined that KLF12 binds to the promoter region of LncRNA-PACERR in TAMs by ChIP assay (Fig. 6a). Then we predicted the binding sites of the promoter region of LncRNA-PACERR based on the motif of KLF12 and found that KLF12 binds to the -302- + 100 bp upstream of LncRNA-PACERR (Fig. 6b–d and Additional file 6: Fig. S10a). To further clarify the specific binding sites of KLF12, the region potentially bound to the LncRNA-PACERR promoter was added into luciferase reporter plasmids and was mutated at each of the 2 putative binding sites respectively. Next, pcDNA-KLF12 and Luc-WT/Luc-Mut1/Luc-Mut2/Luc-Mut3 were co-transfected in HEK-293 T cells. We confirmed that the exact site of KLF12 binding to LncRNA-PACERR was -348 ~ -340 bp. Given that most transcription factors regulate gene transcription by affecting histone modifications, we used ChIP assays to determine that KLF12 increased enrichment of H3K27ac at TSS of LncRNA-PACERR in TAMs (Fig. 6f and Additional file 6: Fig. S10b). Therefore, we considered whether KLF12 would recruit the classical histone acetyltransferase-EP300. This conjecture was verified by co-IP assay (Fig. 6g). Interestingly, based on previous studies reporting that LncRNAs can form complexes with transcription factors to exert transcriptional regulation [43], we hypothesized that KLF12 would bind to LncRNA-PACERR in nucleus. We next performed RNA pull-down and RIP assays and validated this interaction (Fig. 6h, i). To further confirm the exact binding site of KLF12, we predicted RNA-binding sequences by catRAID and constructed Flag-tagged deletion mutant plasmids of KLF12 (Additional file 6: Fig. S10c, d). RIP results showed that 311aa-399aa of KLF12 binds to LncRNA-PACERR. Finally, we observed that LncRNA-PACERR is required for KLF12 to recruit EP300 by ChIP assay (Additional file 6: Fig. S10e). To sum up, our data indicated that LncRNA-PACERR is also directly transcriptionally regulated by KLF12.

### LncRNA-PACERR exerts oncogenic effects by cooperating with IGF2BP2 in TAMs

**Fig. 7:**
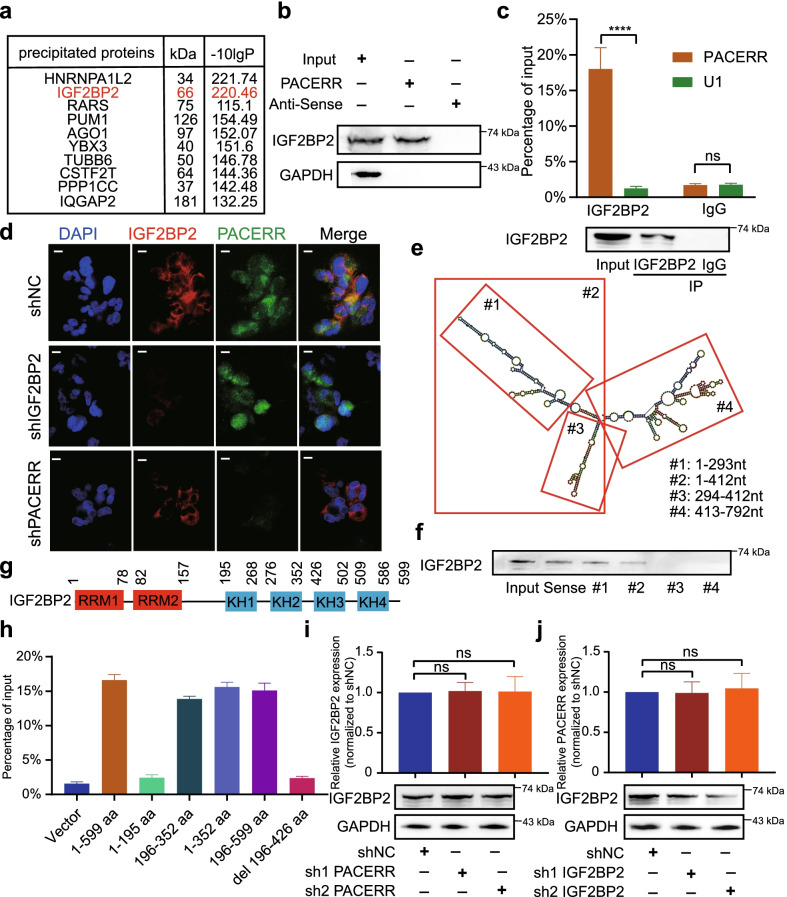
LncRNA-PACERR directly binds with IGF2BP2 in TAMs. **A** Visualization of protein bands by biotin-labelled LncRNA-PACERR RNA probes incubated with total protein extracts from THP-1 derived TAMs. **B** Immunoblotting to determine the specific association of IGF2BP2 with biotinylated LncRNA-PACERR. **C** qRT-PCR analysis of LncRNA-PACERR enriched by IGF2BP2 in THP-1 derived TAMs (top). Immunoblot of IGF2BP2 is shown (bottom). IP, immunoprecipitation. **D** Images showing the colocalization of LncRNA-PACERR and IGF2BP2 in the IGF2BP2 KD and LncRNA-PACERR KD THP-1 derived TAMs. Scale bars: 20 μm. **E** Secondary structure of LncRNA-PACERR analysed by RNAfold web server and deletion mapping of biotinylated LncRNA-PACERR motifs, as indicated. The red boxes represent the remaining fragments of LncRNA-PACERR, with the corresponding number label in the corner. **F** Immunoblot showing the association of IGF2BP2 with biotinylated LncRNA-PACERR RNA strands and the above-mentioned biotinylated LncRNA-PACERR motifs. **G** Schematic structures showing six domains in IGF2BP2. **H** RIP analysis for LncRNA-PACERR enrichment in HEK293T cells transfected with the FLAG-tagged full-length or truncated IGF2BP2 constructs (*n* = 3). aa: amino acid. **I** qRT-PCR analysis of IGF2BP2 mRNA levels (top) and immunoblot of IGF2BP2 (bottom) in the LncRNA-PACERR KD cells (*n* = 3). **J** qRT-PCR analysis of LncRNA-PACERR levels (top) and immunoblot of IGF2BP2 (bottom) in the IGF2BP2 KD cells (*n* = 3). The results are presented as the mean ± SEM. *P < 0.05; **P < 0.01; ***P < 0.001; ****P < 0.0001, *ns* not significant

Since it was previously reported that lncRNA can function in the cytoplasm by binding to specific proteins [21, 44], we performed RNA pull-down assays and mass spectrometry to screen for an m6A-associated protein-IGF2BP2 that interacts with LncRNA-PACERR (Fig. 7a and Additional file 6: Fig. S11a). Then, we further validated IGF2BP2 as an RNA-binding protein for LncRNA-PACERR (Fig. 7b and Additional file 6: Fig. S11b). In addition, RIP experiments confirmed the relationship between IGF2BP2 and LncRNA-PACERR (Fig. 7c). Furthermore we supported their interaction by co-localization analysis of LncRNA-PACERR FISH and IGF2BP2 in THP-1-drived TAMs (Fig. 7d). To explore the specific binding regions of LncRNA-PACERR and IGF2BP2, we predicted the binding sites of LncRNA-PACERR and IGF2BP2 based on catRAPID and constructed truncated plasmids according to the corresponding regions (Additional file 6: Fig. S12a). Next, we determined that the 1-293nt region of LncRNA-PACERR interacts with IGF2BP2 by RNA pull-down and western blot assays (Fig. 7e, f). According to the previous literature, IGF2BP2 possesses two RNA recognition molecule (RRM) structural domains and four KH homologous structural domains [45]. We constructed HEK-293 T cells which contained FLAG-tagged truncated and full-length IGF2BP2 and found that the KH1 and KH2 structural domains are decisive for the recruitment of LncRNA-PACERR by using RIP-qPCR assays (Fig. 7g, h and Additional file 6: Fig. S12b). Interestingly, we found that LncRNA-PACERR and IGF2BP2 do not change each other's expression levels by qPCR, western blot, FISH and IF assays (Fig. 7d, j, k, Additional file 6: Fig. S7e and Fig. S12c-f). These results suggested a physical binding interaction of LncRNA-PACERR with IGF2BP2.

### LncRNA-PACERR increased mRNA stability of KLF12 and c-myc by cooperating with IGF2BP2

**Fig. 8:**
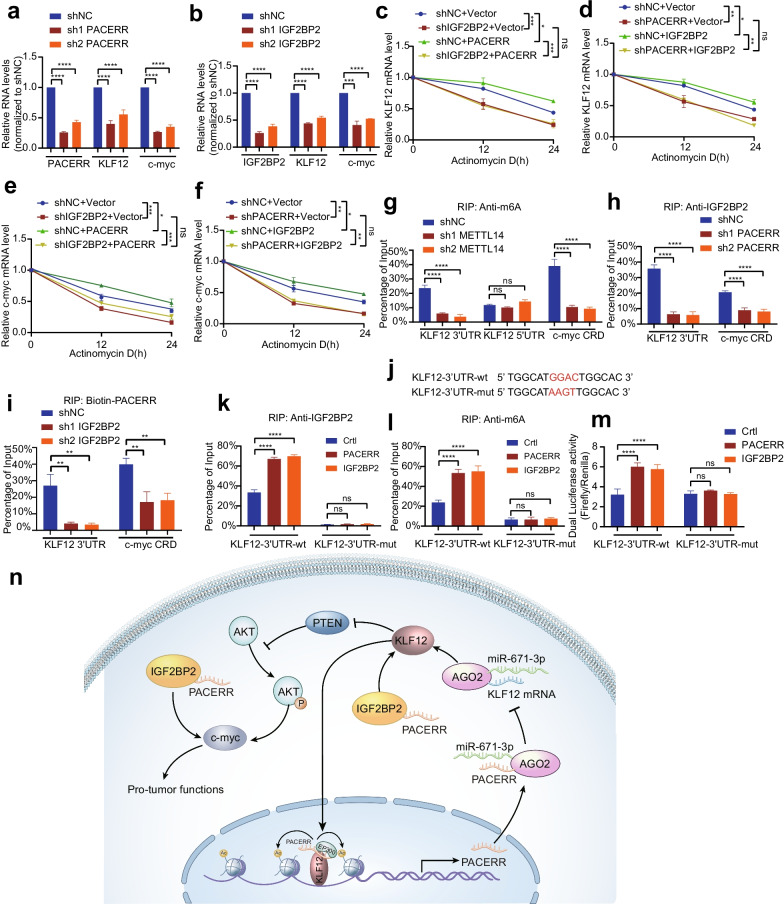
LncRNA-PACERR cooperates with IGF2BP2 to regulate KLF12 and c-myc in an m6A-dependent manner. **A**, **B** qRT-PCR analysis of the LncRNA-PACERR, KLF12 and c-myc transcript levels in the LncRNA-PACERR KD (A) and IGF2BP2 KD (B) THP-1 derived TAMs (*n* = 3). **C**, **D** Half-life of KLF12 after treatment with 5 μmol/L actinomycin D for the indicated times in the IGF2BP2 KD THP-1 derived TAMs with ectopically expressed LncRNA-PACERR (**C**) and in the LncRNA-PACERR KD THP-1 derived TAMs with ectopically expressed IGF2BP2 (**D**). **E**, **F** Falf-life of c-myc after treatment with 5 μmol/L actinomycin D for the indicated times in the IGF2BP2 KD THP-1 derived TAMs with ectopically expressed LncRNA-PACERR (**E**) and in the LncRNA-PACERR KD THP-1 derived TAMs with ectopically expressed IGF2BP2 (**F**). **G** RIP qRT-PCR showing the enrichment of m6A modification in the KLF12 3′ UTR/5′ UTR and c-myc CRD regions in the METTL14 KD THP-1 derived TAMs (*n* = 3). **H**, **I** RIP qRT-PCR detecting the enrichment of IGF2BP2 (**H**) and biotin-labelled LncRNA-PACERR (**I**) in the KLF12 3′ UTR and c-myc CRD in LncRNA-PACERR KD (H) and IGF2BP2 KD (I) THP-1 derived TAMs (*n* = 3). **J** Schematic representation of wild-type (WT) and mutated (MUT; GGAC to AAGT) KLF12 3′ UTR of the pmirGLO vector. **K**, **L** RIP qRT-PCR detection of the enrichment of IGF2BP2 (**K**) and m6A (**L**) in the KLF12 3′ UTR WT and MUT luciferase reporters in the LncRNA-PACERR and IGF2BP2 OE cells (*n* = 3). **M** Relative luciferase activity levels of KLF12 3′ UTR WT and MUT reporters in the LncRNA-PACERR and IGF2BP2 OE cells (*n* = 3). **N** Proposed model demonstrating a positive feedback loop between LncRNA-PACERR and KLF12 in TAMs to promote proliferation and migration in PDAC. The expression of LncRNA-PACERR is activated by EP300-mediated H3K27 acylation. LncRNA-PACERR exerts its pro-tuomour function by regulating miR-671-3p/KLF12/AKT/c-myc axis and sequestering IGF2BP2

Numerous studies have reported that IGF2BP2 acts as an m6A reader that could stabilize a large number of mRNA transcripts, including c-myc and many transcription factors [30, 46]. Meanwhile, with KLF12 and c-myc as downstream targets of LncRNA-PACERR, we were very curious whether LncRNA-PACERR cooperated with IGF2BP2 to regulate the stability of KLF12 and c-myc. To confirm this hypothesis, we first used qPCR and western blot analysis to clarify the positive regulatory effects of LncRNA-PACERR/IGF2BP2 on KLF12 and c-myc expression (Fig. 8a, b and Additional file 6: Fig. S12g-l). To further investigate whether LncRNA-PACERR/IGF2BP2 mediates the stabilization of KLF12 and c-myc, we used actinomycin D to find that IGF2BP2 knockdown abrogated the increased stabilization of KLF12 and c-myc by LncRNA-PACERR overexpression, and LncRNA-PACERR knockdown abolished the half-life of KLF12 and c-myc by IGF2BP2 overexpression (Fig. 8c-f). To further clarify whether KLF12 and c-myc are modified by m6A methylation, we used m6A RIP qRT-PCR assays based on the ‘GGAC’ m6A core motif to show that the m6A methylation of 3′ untranslated region (UTR) of the KLF12 transcript and the coding region instability determinant (CRD) region of the c-myc transcript were decreased in methyltransferase-like 14 (METTL14) knockdown THP-1 cells (Fig. 8g). To exclude the possibility that c-myc and KLF12 may be regulated directly by m6A writers, we found that the expression of Mettl3, Mettl14 and WTAP was not changed after knockdown of LncRNA-PAERR in THP-1 derived TAMs (Additional file 6: Fig. S13a). To explore whether LncRNA-PACERR affects the binding of IGF2BP2 to the m6A-modified regions of KLF12 and c-myc, we found that LncRNA-PACERR knockdown significantly attenuated IGF2BP2 binding to the m6A-modified regions of KLF12 and c-myc by RIP-qPCR assays (Fig. 8h, i and Additional file 6: Fig. S13b). Meanwhile the binding of LncRNA-PACERR to the m6A modification region of KLF12 and c-myc was almost abolished by IGF2BP2 knockdown. To further determine that IGF2BP2 is required for LncRNA-PACERR to bind to KLF12 and c-myc, we found that their interaction was significantly attenuated by treatment with proteinase K in TAMs (Additional file 6: Fig. S13c). As previous studies have described the regulation of m6A modification of c-myc by IGF2BP2 in detail [30], we focused on exploring the regulator mechanism of IGF2BP2 to KLF12. Based on the available literature reporting that IGF2BP2-regulated m6A modification regions are mainly at the starting point of the 3'UTR of mRNA, we constructed a luciferase reporter for the 3′ UTR of KLF12 and then validated that IGF2BP2 and m6A were enriched in the wild-type reporter compared to the mutant reporter by RIP-qPCR (Fig. 8j–l and Additional file 6: Fig. S13d, e). In addition, dual luciferase reporter assays showed that LncRNA-PACERR increased luciferase activity in the KLF12-3′ UTR wild-type reporter, and with similar results for IGF2BP2 (Fig. 8m and Additional file 6: Fig. S13f).

These data indicated that LncRNA-PACERR cooperates with IGF2BP2 to enhance the stability of KLF12 and c-myc in TAMs.

## Discussion

Current immunotherapy for pancreatic cancer has been mostly disappointing, and TAMs, as an important component of the pancreatic cancer microenvironment, have become the key object of many studies [47]. Dysregulated expression of lncRNAs is closely associated with cancer development and progression [48]. Therefore, the finding of lncRNAs in TAMs with the function of driving macrophage polarization is essential for comprehensive understanding of the characteristics of the TME. In the present study, we used fresh tissue specimens from 46 PDAC patients and pathological TMAs from 110 patients in our centre to reveal for the first time that the expression of LncRNA-PACERR was significantly upregulated in TAMs and closely associated with poor prognosis of PDAC. Then we clarified the pro-tumour function of LncRNA-PACERR in vitro and in vivo. This result is consistent with previous reports. In terms of molecular mechanisms, we explored the role of LncRNA-PACERR in the cytoplasm and nucleus, respectively. In the cytoplasm, we identified that LncRNA-PACERR was as a ceRNA to sponge miR-671-3p, thereby releasing KLF12 to activate the AKT/c-myc pathway, and was as an m6A-dependent manner to cooperate with IGF2BP2 for stabilizing KLF12 and c-myc. In the nucleus, it is formed a positive feedback loop that KLF12/LncRNA-PACERR complex recruits EP300 to promote transcription of LncRNA-PACERR (Fig. 8n).

The effect of miR-671-3p on tumours is still controversial and mostly based on studies of tumour cells [24, 49]. The role of miR-671-3p has not been reported in TME. In our study, we found that miR-671-3p was significantly downregulated in TAMs, inhibited M2 macrophage polarization and attenuated pancreatic cancer cell proliferation and metastasis by directly targeting KLF12. KLF12 has been extensively studied in pancreatic cancer and can promote pancreatic cancer malignant progression via AKT/c-myc axis, which has not been reported in TAMs. This axis was verified in our study.

In addition to exploring the downstream pathway of LncRNA-PACERR, we also studied the reasons for the upregulation of LncRNA-PACERR in TAMs. We amazedly found that KLF12 is directly enriched at the promoter region of LncRNA-PACERR and recruits EP300 after forming a complex with LncRNA-PACERR in the nucleus. This positive feedback loop mechanism was extended insights into epigenetic modifications of LncRNA-PACERR in TAMs.

It has been reported that IGF2BP2 could represent an independent predictor of pancreatic cancer [50]. The mechanism of IGF2BP2 in macrophages from PDAC has not been elucidated. We show that the ability of LncRNA-PACERR to drive pancreatic cancer is dependent on IGF2BP2. Interestingly, LncRNA-PACERR cooperates with IGF2BP2 to increase the expression of KLF12 and c-myc. However, the exact sites at which they regulate KLF12 and c-myc are different. The well-characterized function of LncRNA-PACERR on IGF2BP2 needs to be further investigated. Although many studies have reported the importance of lncRNAs in participating in the regulation of IGF2BP2 target stability [51, 52]. However, it is not clear how lncRNAs interact with IGF2BP2. Our findings not only confirm that LncRNA-PACERR can bind to IGF2BP2 and enhance its function, but also suggest a more precise mechanism by which the partial secondary structure of LncRNA-PACERR interacts with the KH1 and KH2 structural domains of IGF2BP2 protein and cooperates in an m6A-dependent manner to stabilize KLF12 and c-myc, probably facilitating PDAC pathogenesis.

However, our studies on the mechanistic aspects are based on in vitro TAMs models, which cannot simulate the complete tumour microenvironment. Therefore, this result would require future exploration and validation through clinical trials. Simultaneously, whether LncRNA-PACERR, a key factor in promoting pancreatic cancer, has the opportunity to become a drug for immunotherapy of PDAC and how to target specific TAMs across the dense connective tissue of PDAC is the new direction of our subsequent research.

## Conclusion

We proved that LncRNA-PACERR promotes malignant progression of pancreatic cancer through two pathways: as ceRNA sponging miR-671-3p to activate the KLF12/AKT/c-myc pathway and interacting with IGF2BP2 to enhance KLF12 and c-myc stability. Meanwhile the KLF12-transcribed LncRNA-PACERR interacts directly with KLF12 and the KLF12/PACERR complex activates LncRNA-PACERR transcription by recruiting EP300, thereby promoting M2 polarization and pro-tumour function in TAMs.

## Supplementary Information

**Additional file 1**. Clinicopathologic characteristics of PDAC patients from Ruijin Hospital in a cDNA microarray of Macrophages.

**Additional file 2**. Clinicopathologic characteristics of PDAC patients from Ruijin Hospital in a tissue array.

**Additional file 3**.  Sequences targeting related genes.

**Additional file 4**. All primer sequences used in the qPCR process.

**Additional file 5**. Antibodies used for western blotting.

**Additional file 6**. Supplementary Figures: 1–13.

**Additional file 7**. Supplementary methods.

**Additional file 8**. Supplementary Figure legends.

## Data Availability

All relevant datasets supporting the key findings of this study are available within the article and its supplementary files or from the corresponding author on reasonable request. The RNA pull-down mass spectrometry data have been submitted to iProX (http://www.iprox.cn) under Accession Number: IPX0003961000.

## Abbreviations

IGF2BP2: Insulin-like growth factor 2 mRNA-binding protein 2
KLF12: Kruppel like factor 12
LncRNA-PACERR: Long non-coding RNA PTGS2 antisense NFKB1 complex-mediated expression regulator RNA
M1-NTRMs: M1 macrophages in normal tissue-resident macrophages
Mut: Mutation
PDAC: Pancreatic ductal adenocarcinoma
RIP: RNA immunoprecipitation
TAMs: Tumour-associated macrophages
TMAs: Tissue microarrays
WT: Wild-type
