# Supplementary material for: LncRNA-PACERR induces pro-tumour macrophages via interacting with miR-671-3p and m6A-reader IGF2BP2 in pancreatic ductal adenocarcinoma
Source: J Hematol Oncol. 2022 May 7;15:52. doi: 10.1186/s13045-022-01272-w (PMC9077921; doi:10.1186/s13045-022-01272-w)
Supplement: Supplementary file 1 — Additional file 1. Clinicopathologic characteristics of PDAC patients from Ruijin Hospital in a cDNA microarray of Macrophages. [file 13045_2022_1272_MOESM1_ESM.docx]

**Table S1 Clinicopathologic characteristics of PDAC patients from Ruijin Hospital in a cDNA microarray of Macrophages.**

| **Characteristics** | **Number of cases** |
| --- | --- |
| **Gender** | |
| Male | 23 |
| Female | 23 |
| **Age (years)** | |
| ≥ 60 | 29 |
| < 60 | 17 |
| **Pathologic stage** | |
| IA  IB | 0  1 |
| IIA | 10 |
| IIB  III  IV | 18  8  9 |
| **T classification** | |
| T1  T2 | 0  1 |
| T3 | 33 |
| T4 | 12 |
| **N classification** | |
| N0  N1 | 16  30 |
| **M classification** | |
| M0  M1 | 37  9 |
