# Supplementary material for: LncRNA-PACERR induces pro-tumour macrophages via interacting with miR-671-3p and m6A-reader IGF2BP2 in pancreatic ductal adenocarcinoma
Source: J Hematol Oncol. 2022 May 7;15:52. doi: 10.1186/s13045-022-01272-w (PMC9077921; doi:10.1186/s13045-022-01272-w)
Supplement: Supplementary file 2 — Additional file 2. Clinicopathologic characteristics of PDAC patients from Ruijin Hospital in a tissue array. [file 13045_2022_1272_MOESM2_ESM.docx]

**Table S2 Clinicopathologic characteristics of PDAC patients from Ruijin Hospital in a tissue array.**

| **Characteristics** | **Number of cases** |
| --- | --- |
| **Gender** | |
| Male | 65 |
| Female | 45 |
| **Age (years)** | |
| ≥ 60 | 72 |
| < 60 | 38 |
| **AJCC stage** | |
| IA | 7 |
| IB  IIA | 25  10 |
| IIB | 38 |
| III  IV | 22  8 |
| **T classification** | |
| T1 | 12 |
| T2 | 62 |
| T3  T4 | 18  18 |
| **N classification** | |
| N0 | 51 |
| N1 | 47 |
| Data missing | 12 |
| **M classification** | |
| M0 | 103 |
| M1 | 7 |
| **Survival status** | |
| Dead | 86 |
| Live | 24 |
