# Supplementary material for: LncRNA-PACERR induces pro-tumour macrophages via interacting with miR-671-3p and m6A-reader IGF2BP2 in pancreatic ductal adenocarcinoma
Source: J Hematol Oncol. 2022 May 7;15:52. doi: 10.1186/s13045-022-01272-w (PMC9077921; doi:10.1186/s13045-022-01272-w)
Supplement: Supplementary file 6 — Additional file 6. Supplementary Figures: 1–13. [file 13045_2022_1272_MOESM6_ESM.pdf]

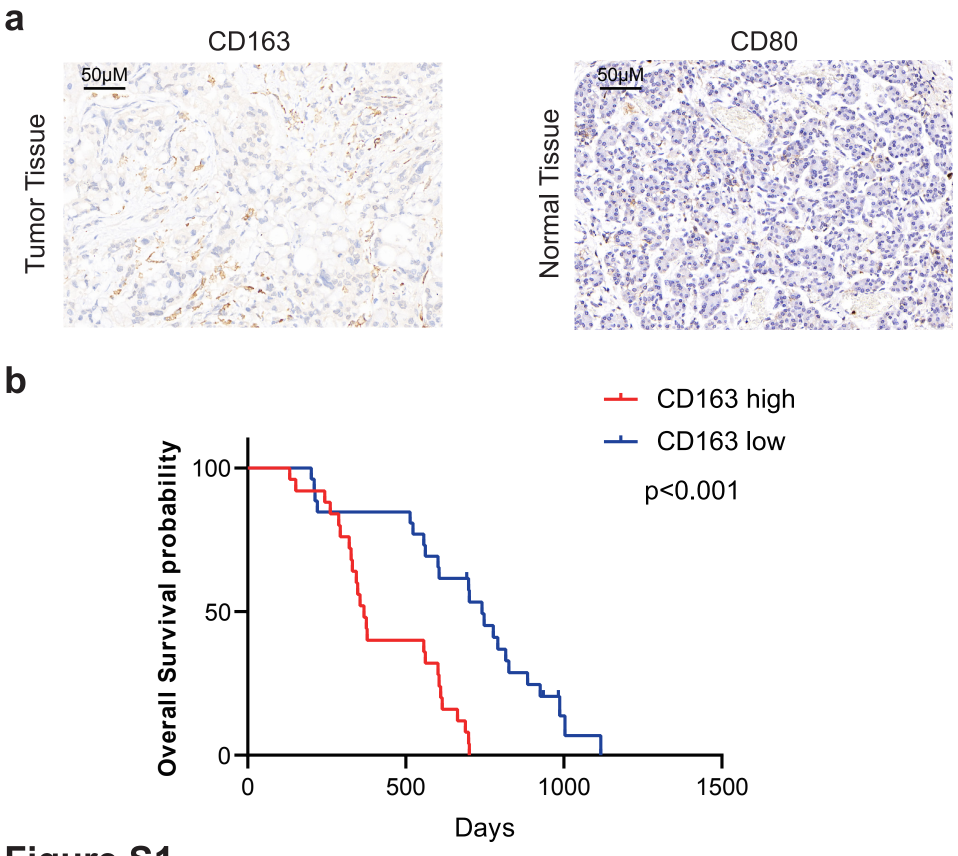
a
CD163
CD80
50μM
50μM
Tumor Tissue
Normal Tissue
b
CD163 high
CD163 low
p<0.001
Overall Survival probability
100
50
0
0
500
1000
1500
Days

Figure S1

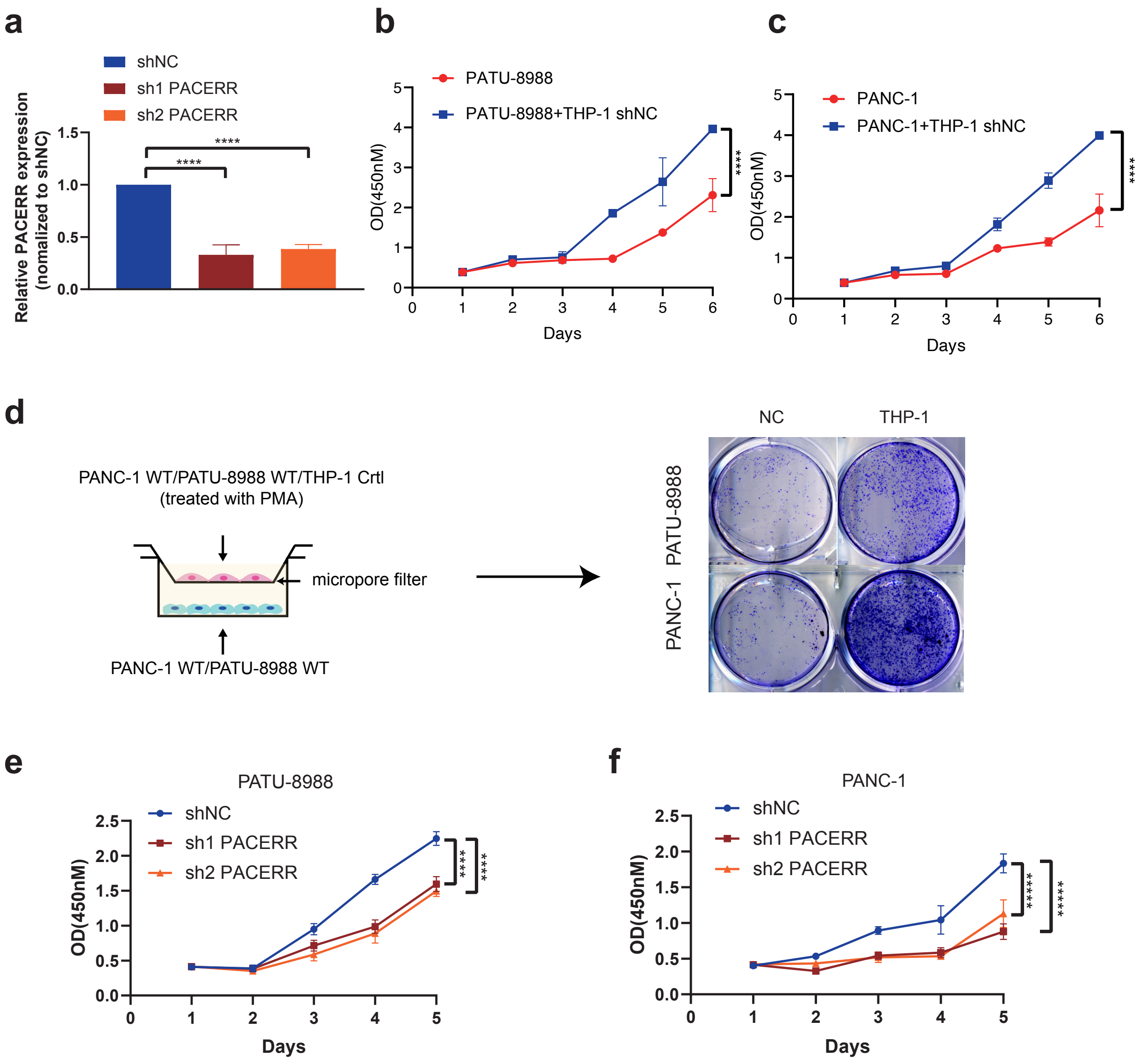
a
shNC
sh1 PACERR
sh2 PACERR
Relative PACERR expression
(nomalized to shNC)
****
****
b
PATU-8988
PATU-8988+THP-1 shNC
OD(450nM)
Days
****
c
PANC-1
PANC-1+THP-1 shNC
OD(450nM)
Days
****
d
PANC-1 WT/PATU-8988 WT/THP-1 Crtl
(treated with PMA)
micropore filter
PANC-1 WT/PATU-8988 WT
NC
THP-1
PATU-8988
PANC-1
e
PATU-8988
shNC
sh1 PACERR
sh2 PACERR
OD(450nM)
Days
****
****
f
PANC-1
shNC
sh1 PACERR
sh2 PACERR
OD(450nM)
Days
*****
*****

Figure S2

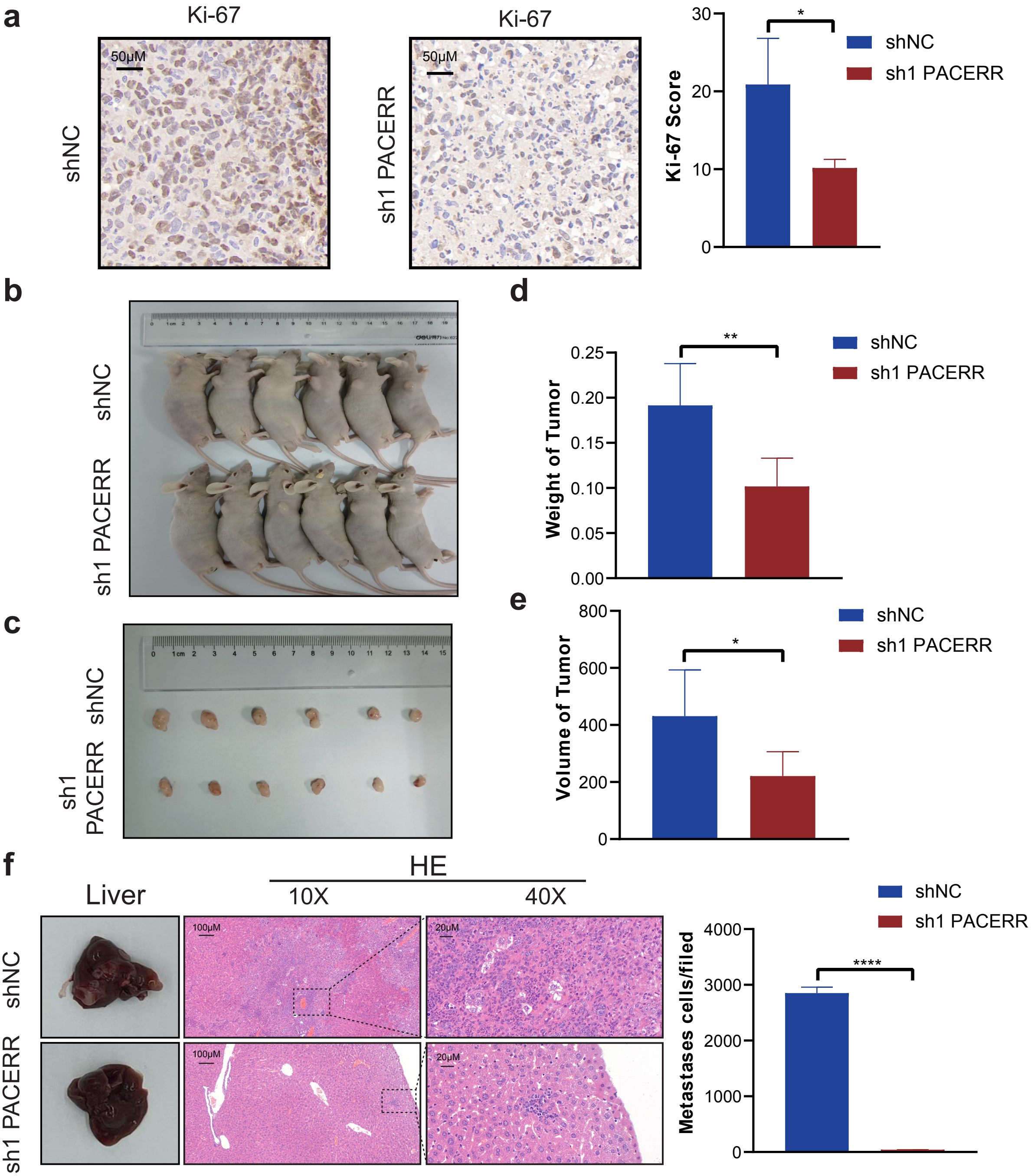

a
Ki-67
Ki-67
shNC
sh1 PACERR
50μM
50μM
Ki-67 Score
*
shNC
sh1 PACERR
b
shNC
sh1 PACERR
c
shNC
sh1 PACERR
d
Weight of Tumor
**
shNC
sh1 PACERR
e
Volume of Tumor
*
shNC
sh1 PACERR
f
Liver
HE
10X
40X
shNC
sh1 PACERR
100μM
20μM
100μM
20μM
Metastases cells/filed
****
shNC
sh1 PACERR

Figure S3

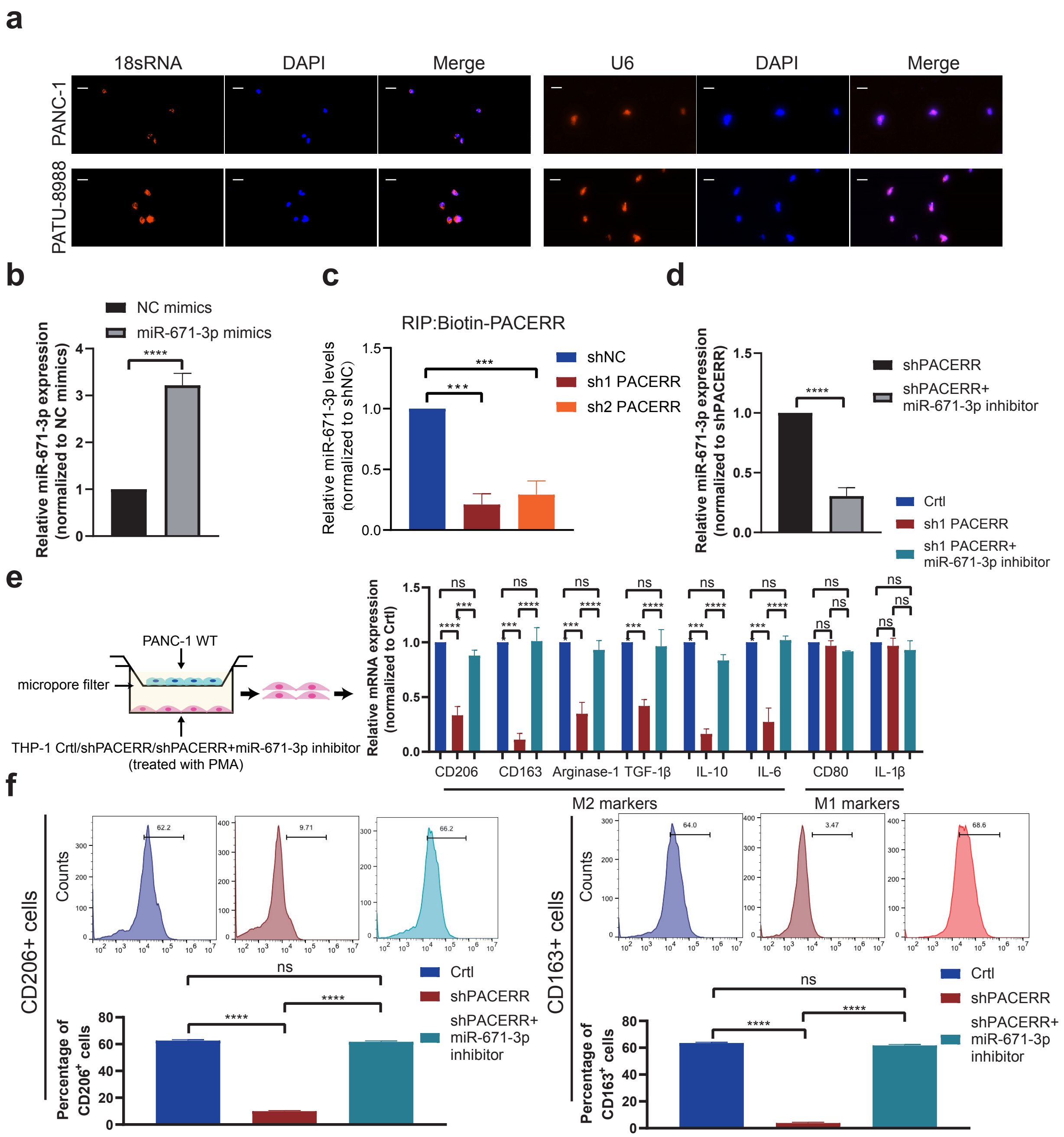

a
18sRNA
DAPI
Merge
U6
DAPI
Merge
PANC-1
PATU-8988
b
NC mimics
miR-671-3p mimics
Relative miR-671-3p expression (normalized to NC mimics)
c
RIP:Biotin-PACERR
shNC
sh1 PACERR
sh2 PACERR
Relative miR-671-3p levels (normalized to shNC)
d
shPACERR
shPACERR+ miR-671-3p inhibitor
Relative miR-671-3p expression (normalized to shPACERR)
e
PANC-1 WT
micropore filter
THP-1 Crtl/shPACERR/shPACERR+miR-671-3p inhibitor
(treated with PMA)
Crtl
sh1 PACERR
sh1 PACERR+ miR-671-3p inhibitor
Relative mRNA expression (normalized to Crtl)
CD206
CD163
Arginase-1
TGF-1β
IL-10
IL-6
CD80
IL-1β
M2 markers
M1 markers
f
CD206+ cells
CD163+ cells
Counts
Crtl
shPACERR
shPACERR+ miR-671-3p inhibitor
Percentage of CD206+ cells
Percentage of CD163+ cells

Figure S4

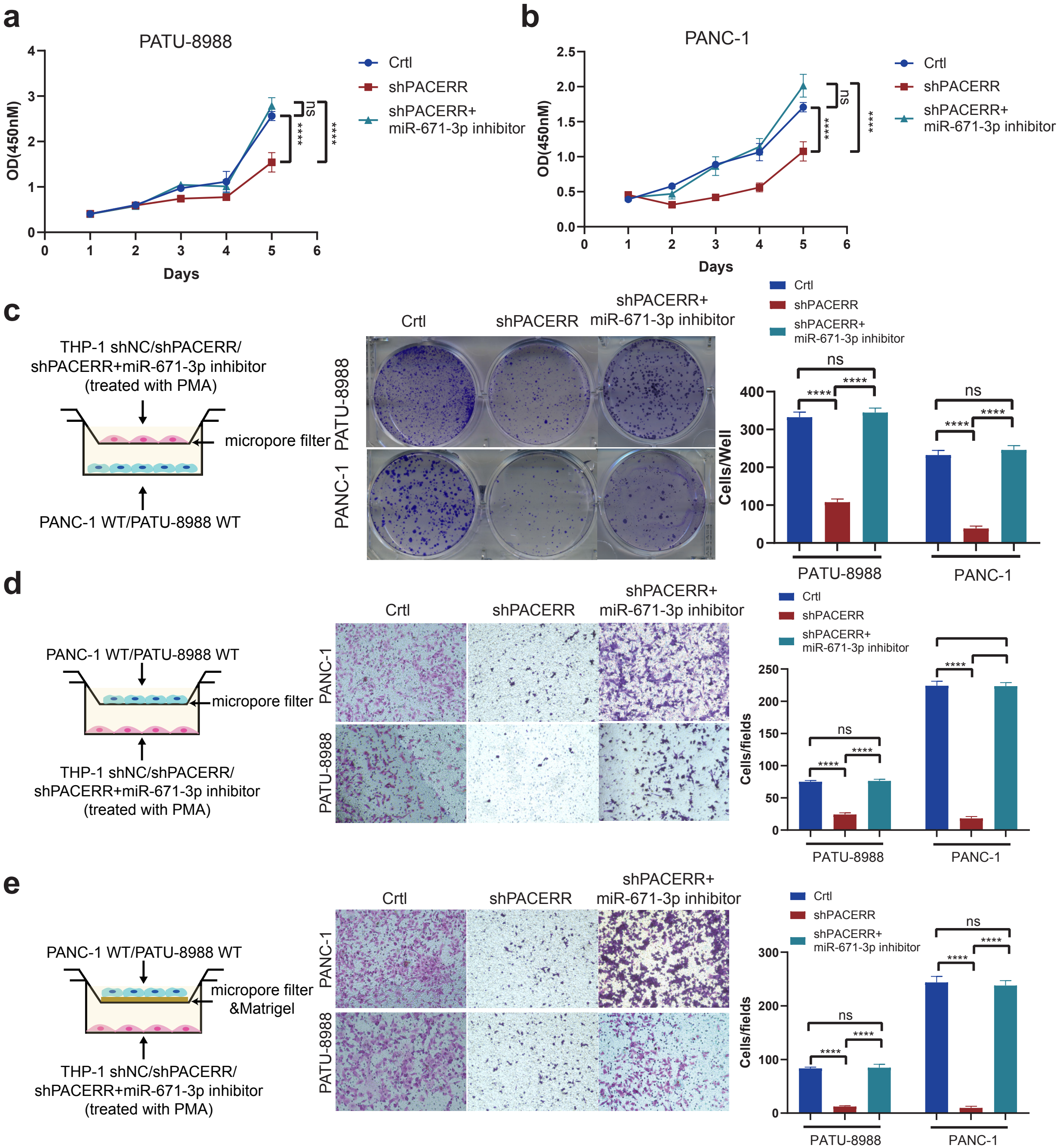
a
PATU-8988
OD(450nM)
Days
Crtl
shPACERR
shPACERR+
miR-671-3p inhibitor
ns
****
****
b
PANC-1
OD(450nM)
Days
Crtl
shPACERR
shPACERR+
miR-671-3p inhibitor
ns
****
****
c
THP-1 shNC/shPACERR/
shPACERR+miR-671-3p inhibitor
(treated with PMA)
micropore filter
PANC-1 WT/PATU-8988 WT
Crtl
shPACERR
shPACERR+
miR-671-3p inhibitor
PATU-8988
PANC-1
Cells/Well
ns
****
PATU-8988
PANC-1
d
PANC-1 WT/PATU-8988 WT
micropore filter
THP-1 shNC/shPACERR/
shPACERR+miR-671-3p inhibitor
(treated with PMA)
Cells/fields
e
PANC-1 WT/PATU-8988 WT
micropore filter
&Matrigel
THP-1 shNC/shPACERR/
shPACERR+miR-671-3p inhibitor
(treated with PMA)
Cells/fields

Figure S5

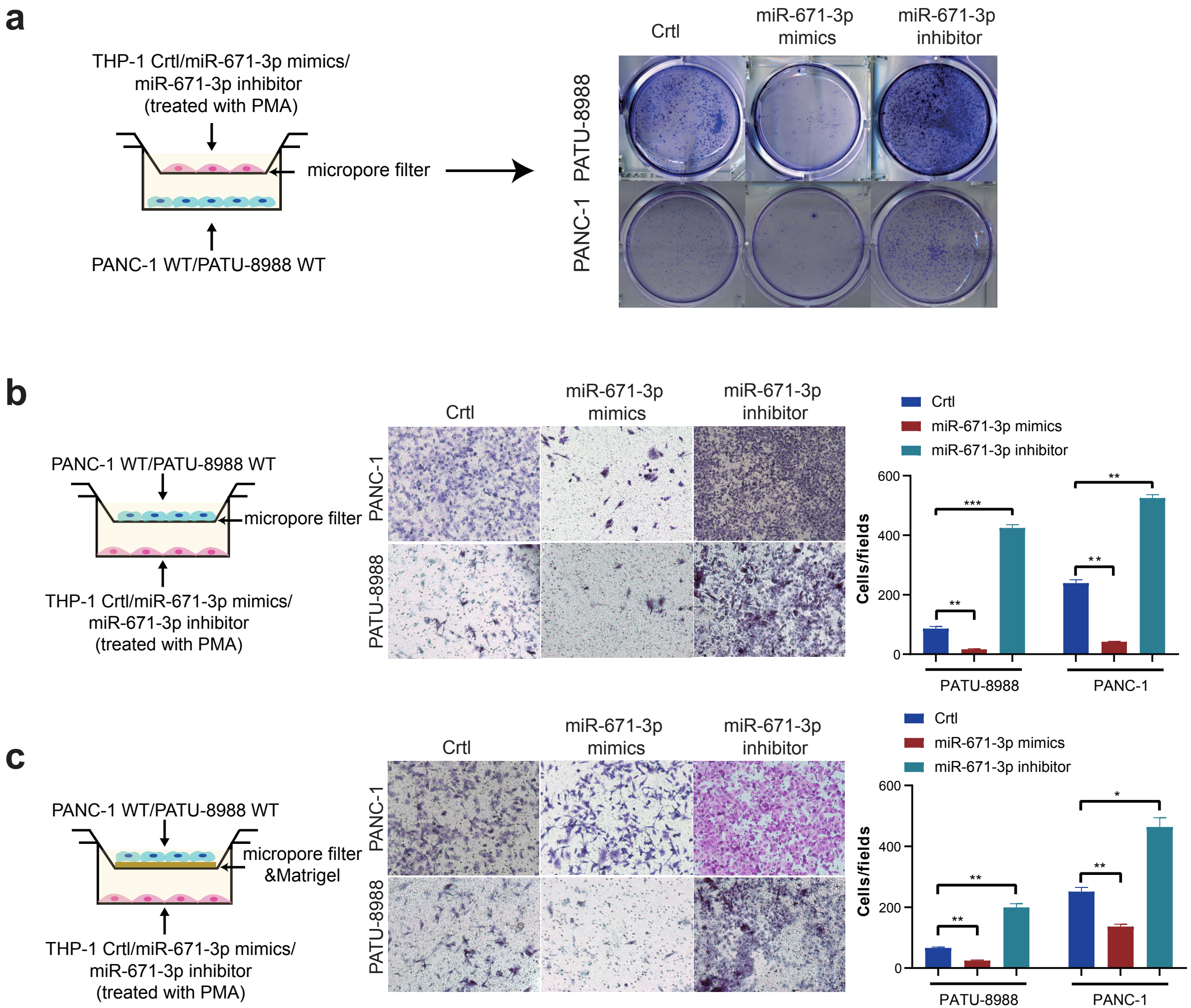
a
THP-1 Crtl/miR-671-3p mimics/
miR-671-3p inhibitor
(treated with PMA)
micropore filter
PANC-1 WT/PATU-8988 WT
Crtl
miR-671-3p mimics
miR-671-3p inhibitor
PATU-8988
PANC-1
b
PANC-1 WT/PATU-8988 WT
micropore filter
THP-1 Crtl/miR-671-3p mimics/
miR-671-3p inhibitor
(treated with PMA)
Crtl
miR-671-3p mimics
miR-671-3p inhibitor
PANC-1
PATU-8988
Crtl
miR-671-3p mimics
miR-671-3p inhibitor
Cells/fields
600
400
200
0
***
**
**
**
PATU-8988
PANC-1
c
PANC-1 WT/PATU-8988 WT
micropore filter
&Matrigel
THP-1 Crtl/miR-671-3p mimics/
miR-671-3p inhibitor
(treated with PMA)
Crtl
miR-671-3p mimics
miR-671-3p inhibitor
PANC-1
PATU-8988
Crtl
miR-671-3p mimics
miR-671-3p inhibitor
Cells/fields
600
400
200
0
**
**
*
**
PATU-8988
PANC-1

Figure S6

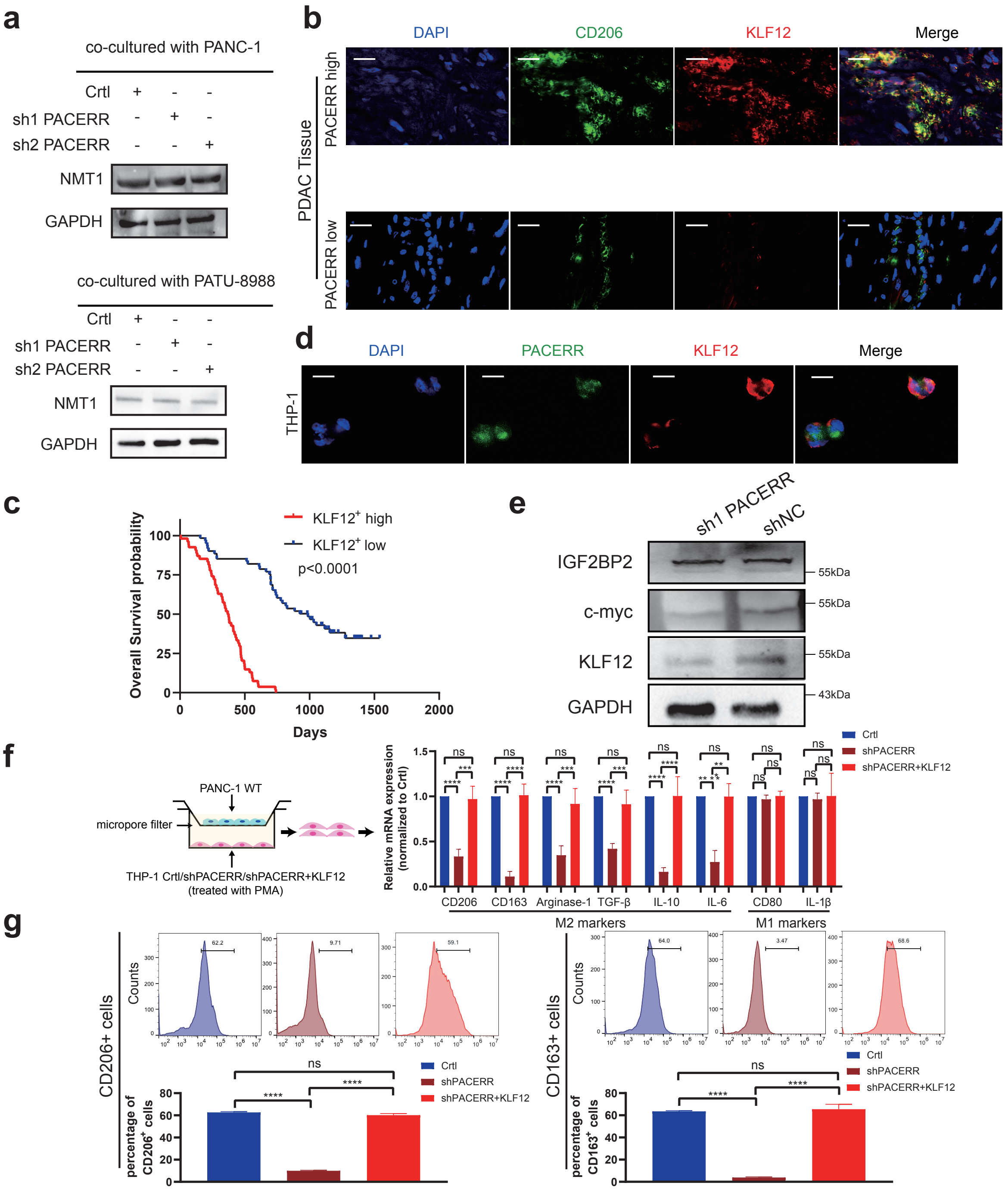

a
co-cultured with PANC-1
Crtl
sh1 PACERR
sh2 PACERR
NMT1
GAPDH
co-cultured with PATU-8988
Crtl
sh1 PACERR
sh2 PACERR
NMT1
GAPDH
b
DAPI
CD206
KLF12
Merge
PDAC Tissue
PACERR high
PACERR low
d
DAPI
PACERR
KLF12
Merge
THP-1
c
Overall Survival probability
KLF12+ high
KLF12+ low
p<0.0001
Days
e
sh1 PACERR
shNC
IGF2BP2
c-myc
KLF12
GAPDH
55kDa
55kDa
55kDa
43kDa
f
PANC-1 WT
micropore filter
THP-1 Crtl/shPACERR/shPACERR+KLF12
(treated with PMA)
Relative mRNA expression
(normalized to Crtl)
Crtl
shPACERR
shPACERR+KLF12
CD206
CD163
Arginase-1
TGF-β
IL-10
IL-6
CD80
IL-1β
M2 markers
M1 markers
g
CD206+ cells
Counts
percentage of CD206+ cells
CD163+ cells
percentage of CD163+ cells
Crtl
shPACERR
shPACERR+KLF12

Figure S7

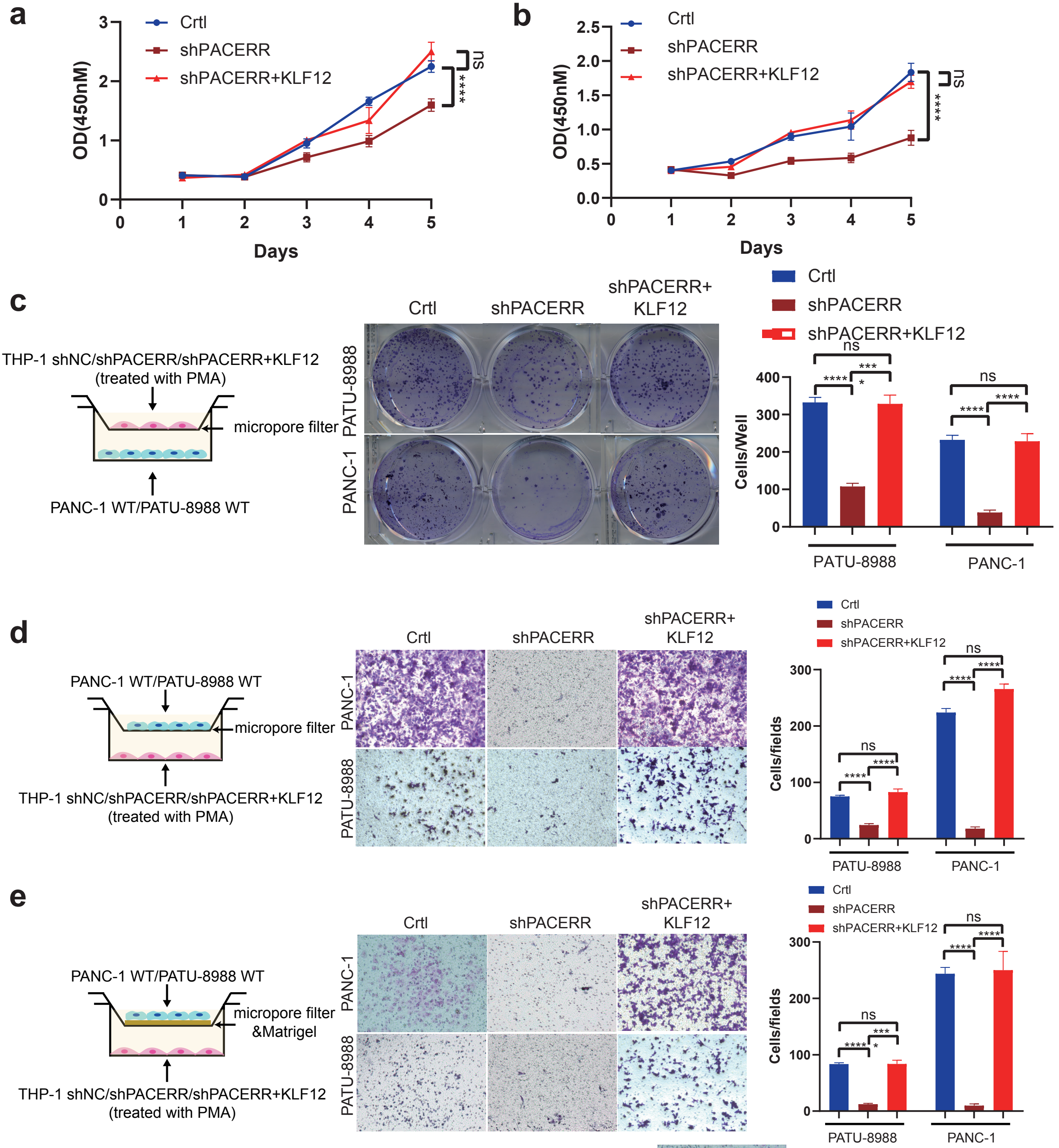
a
OD(450nM)
Days
Crtl
shPACERR
shPACERR+KLF12
ns
****
b
OD(450nM)
Days
Crtl
shPACERR
shPACERR+KLF12
ns
****
c
THP-1 shNC/shPACERR/shPACERR+KLF12
(treated with PMA)
micropore filter
PANC-1 WT/PATU-8988 WT
Crtl
shPACERR
shPACERR+
KLF12
PATU-8988
PANC-1
Cells/Well
ns
d
PANC-1 WT/PATU-8988 WT
micropore filter
THP-1 shNC/shPACERR/shPACERR+KLF12
(treated with PMA)
Cells/fields
e
PANC-1 WT/PATU-8988 WT
micropore filter
&Matrigel
THP-1 shNC/shPACERR/shPACERR+KLF12
(treated with PMA)
Cells/fields

Figure S8

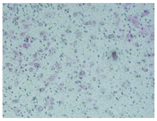

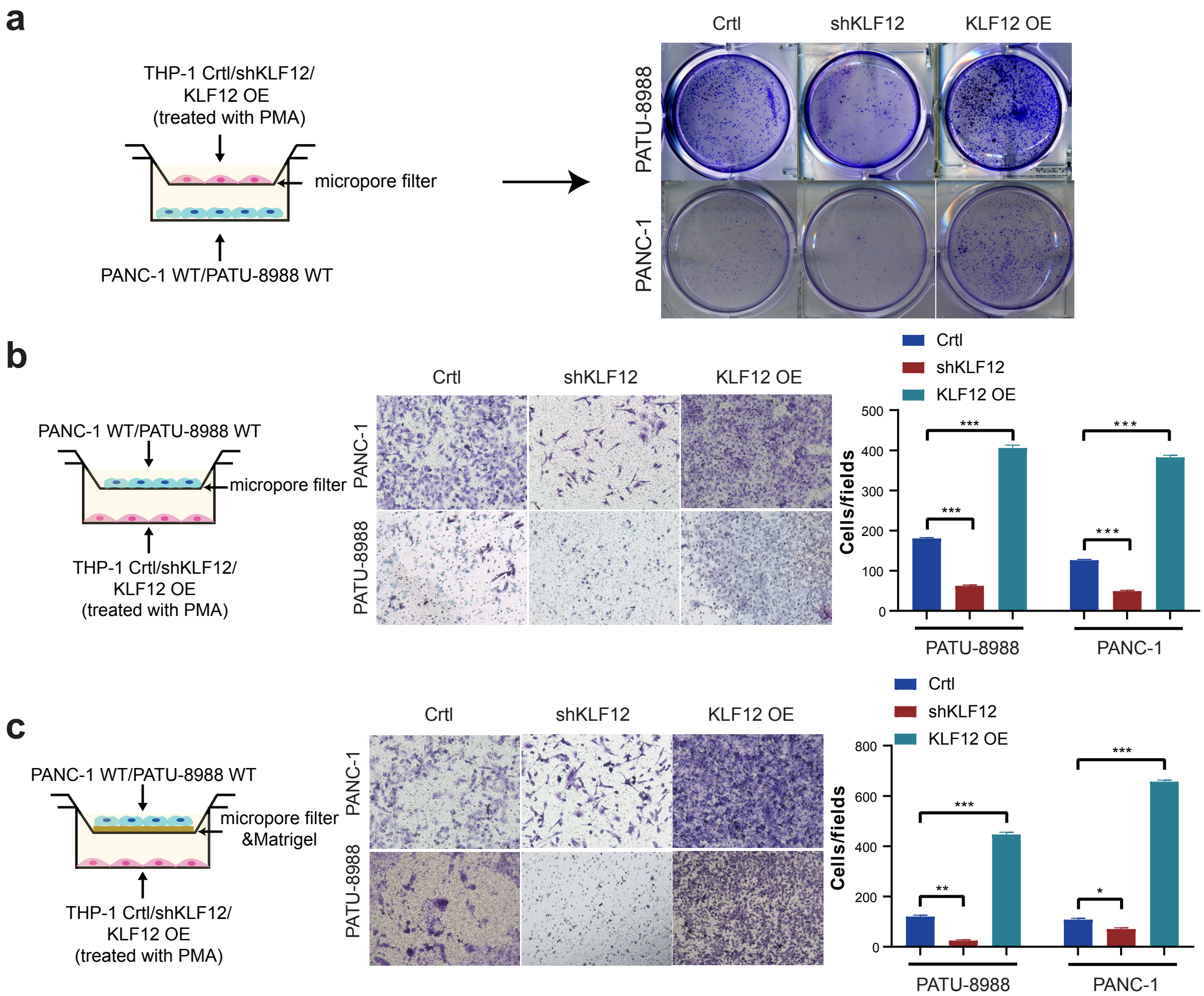
a
THP-1 Crtl/shKLF12/
KLF12 OE
(treated with PMA)
micropore filter
PANC-1 WT/PATU-8988 WT
Crtl
shKLF12
KLF12 OE
PATU-8988
PANC-1
b
PANC-1 WT/PATU-8988 WT
micropore filter
THP-1 Crtl/shKLF12/
KLF12 OE
(treated with PMA)
Crtl
shKLF12
KLF12 OE
PANC-1
PATU-8988
Crtl
shKLF12
KLF12 OE
Cells/fields
500
400
300
200
100
0
***
***
***
***
PATU-8988
PANC-1
c
PANC-1 WT/PATU-8988 WT
micropore filter
&Matrigel
THP-1 Crtl/shKLF12/
KLF12 OE
(treated with PMA)
Crtl
shKLF12
KLF12 OE
PANC-1
PATU-8988
Crtl
shKLF12
KLF12 OE
Cells/fields
800
600
400
200
0
***
***
**
*
PATU-8988
PANC-1

Figure S9

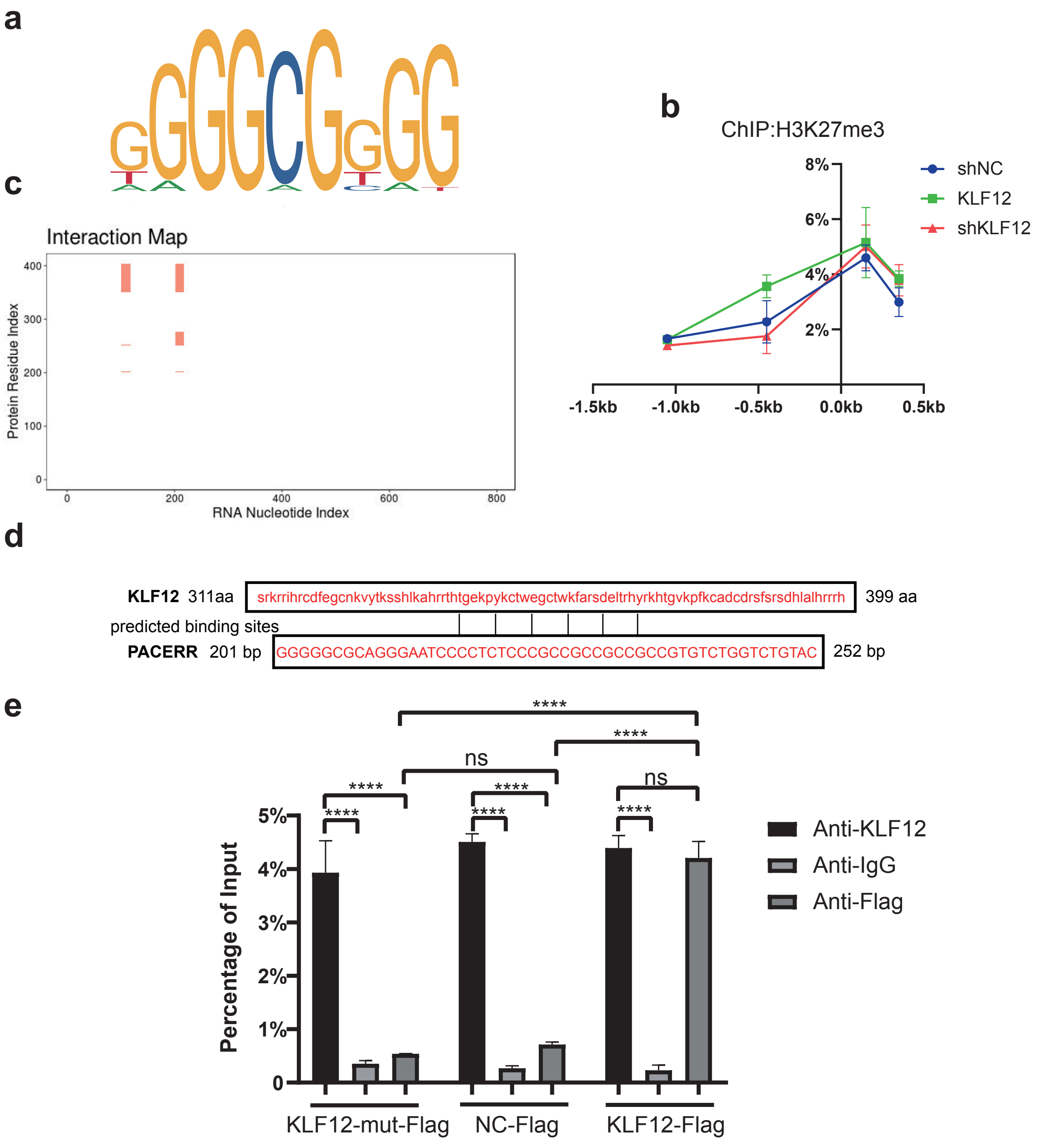
a
c
Interaction Map
Protein Residue Index
400
300
200
100
0
0
200
400
600
800
RNA Nucleotide Index
b
ChIP:H3K27me3
8%
6%
4%
2%
-1.5kb
-1.0kb
-0.5kb
0.0kb
0.5kb
shNC
KLF12
shKLF12
d
KLF12 311aa
srkrrihrcdfegcnkvytksshlkahrrthtgekpykctwegctwkfarsdeltrhyrkhtgvkpfkcadcdrsfsrsdhlalhrrrh
399 aa
predicted binding sites
PACERR 201 bp
GGGGGCGCAGGGAATCCCCTCTCCCGCCGCCGCCGCCGTGTCTGGTCTGTAC
252 bp
e
****
****
ns
ns
****
****
****
****
****
****
Percentage of Input
5%
4%
3%
2%
1%
0
Anti-KLF12
Anti-IgG
Anti-Flag
KLF12-mut-Flag
NC-Flag
KLF12-Flag

Figure S10

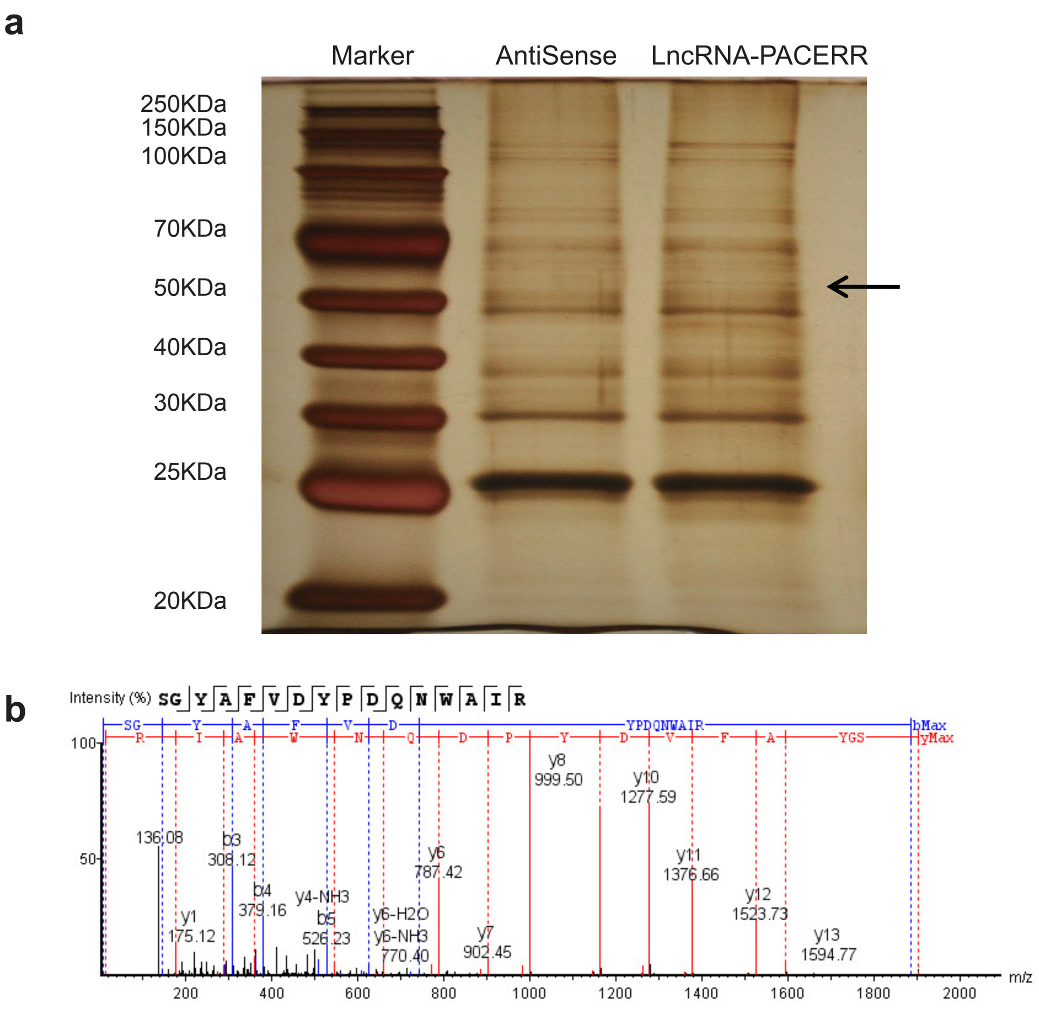
a
Marker
AntiSense
LncRNA-PACERR
250KDa
150KDa
100KDa
70KDa
50KDa
40KDa
30KDa
25KDa
20KDa
b
Intensity (%)
SG Y A F V D Y P D Q N W A I R
SG Y A F V D YPDQNWAIR bMax
R I A W N Q D P Y D V F A YGS yMax
y8 999.50
y10 1277.59
136.08
b3 308.12
y6 787.42
y11 1376.66
b4 379.16
y4-NH3
y12 1523.73
y1 175.12
b5 526.23
y6-H2O
y6-NH3 770.40
y7 902.45
y13 1594.77
100
50
200
400
600
800
1000
1200
1400
1600
1800
2000
m/z

Figure S11

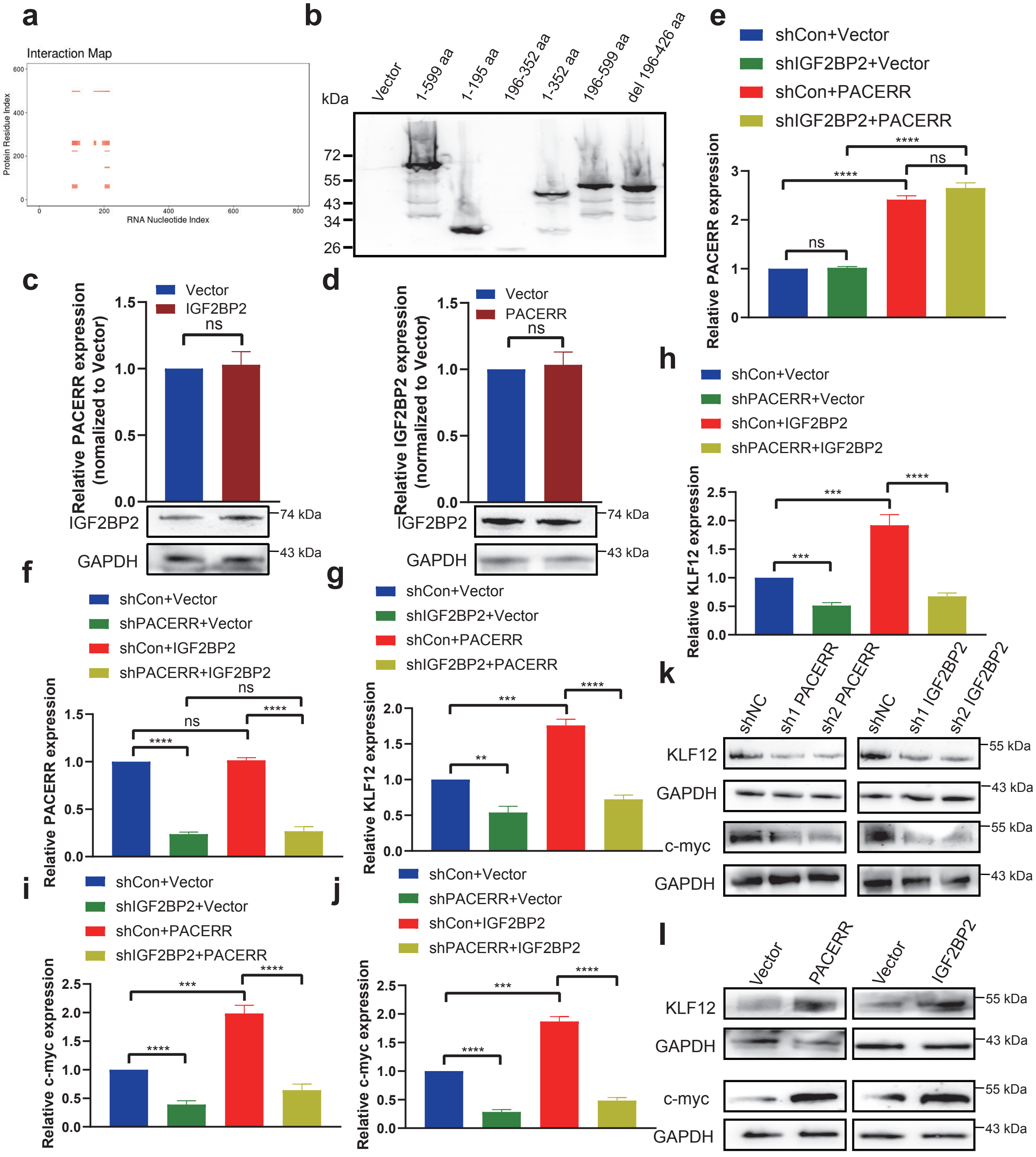

a
Interaction Map
Protein Residue Index
RNA Nucleotide Index
b
kDa
Vector
1-599 aa
1-195 aa
196-352 aa
1-352 aa
196-599 aa
del 196-426 aa
72
55
43
34
26
c
Vector
IGF2BP2
ns
Relative PACERR expression (nomalized to Vector)
IGF2BP2
74 kDa
GAPDH
43 kDa
d
Vector
PACERR
ns
Relative IGF2BP2 expression (normalized to Vector)
IGF2BP2
74 kDa
GAPDH
43 kDa
e
shCon+Vector
shIGF2BP2+Vector
shCon+PACERR
shIGF2BP2+PACERR
Relative PACERR expression
f
shCon+Vector
shPACERR+Vector
shCon+IGF2BP2
shPACERR+IGF2BP2
Relative PACERR expression
g
shCon+Vector
shIGF2BP2+Vector
shCon+PACERR
shIGF2BP2+PACERR
Relative KLF12 expression
h
shCon+Vector
shPACERR+Vector
shCon+IGF2BP2
shPACERR+IGF2BP2
Relative KLF12 expression
i
shCon+Vector
shIGF2BP2+Vector
shCon+PACERR
shIGF2BP2+PACERR
Relative c-myc expression
j
shCon+Vector
shPACERR+Vector
shCon+IGF2BP2
shPACERR+IGF2BP2
Relative c-myc expression
k
shNC
sh1 PACERR
sh2 PACERR
shNC
sh1 IGF2BP2
sh2 IGF2BP2
KLF12
GAPDH
c-myc
GAPDH
55 kDa
43 kDa
l
Vector
PACERR
Vector
IGF2BP2
KLF12
GAPDH
c-myc
GAPDH
55 kDa
43 kDa

Figure S12

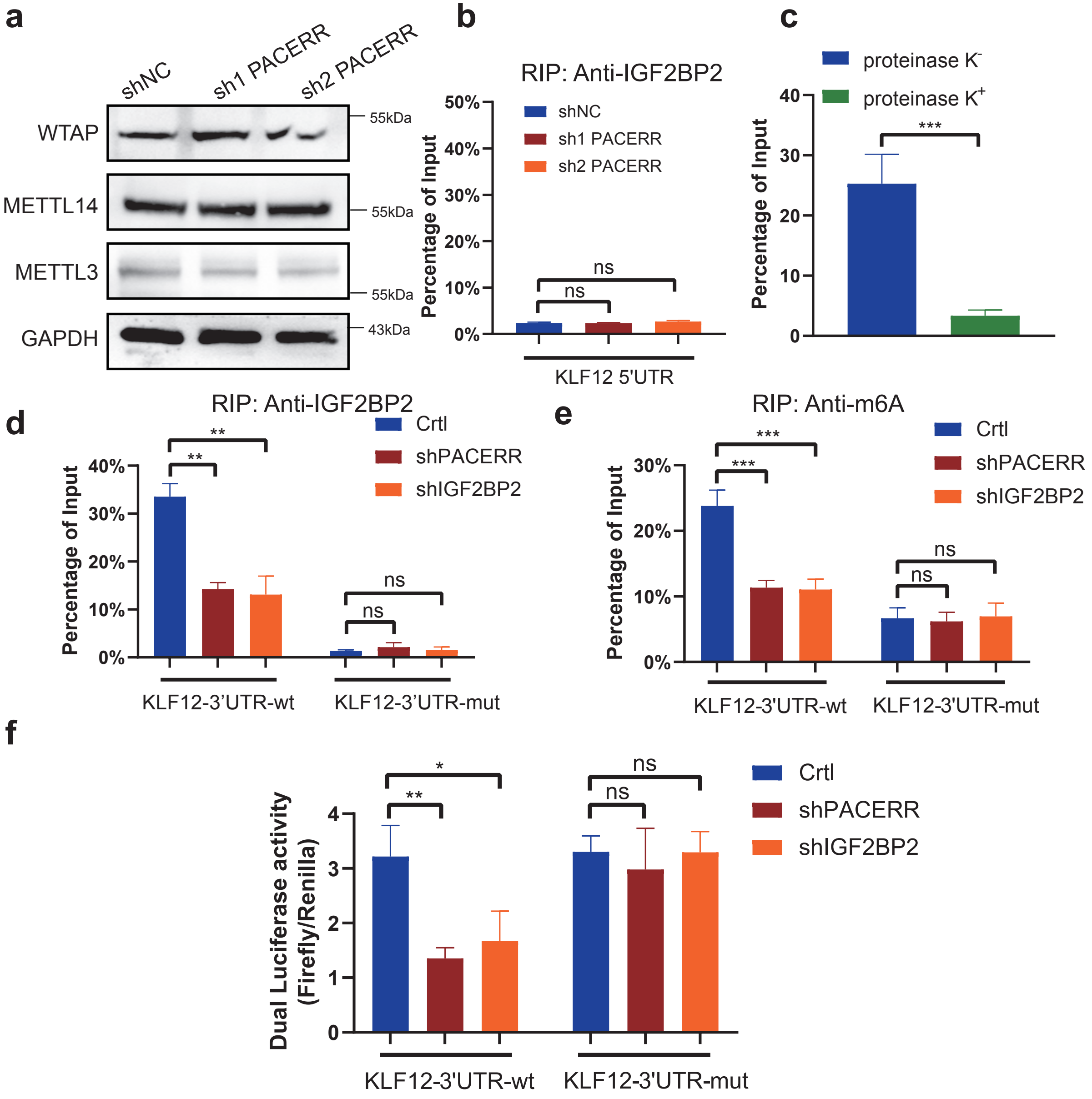
a
shNC
sh1 PACERR
sh2 PACERR
WTAP
METTL14
METTL3
GAPDH
55kDa
55kDa
55kDa
43kDa
b
RIP: Anti-IGF2BP2
shNC
sh1 PACERR
sh2 PACERR
Percentage of Input
50%
40%
30%
20%
10%
0%
ns
ns
KLF12 5'UTR
c
proteinase K-
proteinase K+
Percentage of Input
40
30
20
10
0
***
d
RIP: Anti-IGF2BP2
Crtl
shPACERR
shIGF2BP2
Percentage of Input
40%
30%
20%
10%
0%
**
**
ns
ns
KLF12-3'UTR-wt
KLF12-3'UTR-mut
e
RIP: Anti-m6A
Crtl
shPACERR
shIGF2BP2
Percentage of Input
30%
20%
10%
0%
***
***
ns
ns
KLF12-3'UTR-wt
KLF12-3'UTR-mut
f
Dual Luciferase activity (Firefly/Renilla)
4
3
2
1
0
*
**
ns
ns
Crtl
shPACERR
shIGF2BP2
KLF12-3'UTR-wt
KLF12-3'UTR-mut

Figure S13
