# Supplementary material for: LncRNA-PACERR induces pro-tumour macrophages via interacting with miR-671-3p and m6A-reader IGF2BP2 in pancreatic ductal adenocarcinoma
Source: J Hematol Oncol. 2022 May 7;15:52. doi: 10.1186/s13045-022-01272-w (PMC9077921; doi:10.1186/s13045-022-01272-w)
Supplement: Supplementary file 7 — Additional file 7. Supplementary methods. [file 13045_2022_1272_MOESM7_ESM.docx]

**Supplementary Methods**

**RNA pulldown assay**

Biotin-labeled LncRNA-PACERR and its antisense RNA were transcribed with Biotin RNA Labeling Mix (Roche Diagnostics, Indianapolis, IN, USA) and SP6/T7 RNA polymerase (Roche Diagnostics, USA). After purification, biotinylated RNAs were incubated with Panc1 cell lysates for 1 hour at 4 °C. Streptavidin–agarose beads (Invitrogen, USA) were added to each tube for 1 h at room temperature. Finally, the enriched proteins were subjected to SDS-PAGE separation for mass spectrometry or western blot analysis.

**Flow cytometry**

Cells were resuspended in 50 μL of staining buffer (PBS: FBS=1000:1). Then, 1.5 μL of anti-CD163-PE or 1.5 μL of anti-CD206-PE were added to the reaction for 30 minutes at 4 °C. After thirty minutes, THP-1 were washed twice with staining buffer, and 1% formaldehyde was used for fixation at 4 °C. The data were detected by an FCM (Beckman Coulter)

**Co-IP**

500 μL of IP lysis buffer (Beyotime) with Proteinase Inhibitor (NCM Biotech) was used to lyse 1× 10^7^ cells for 10 minutes on ice. Nuclear extracts were centrifuged at 14000 rpm for 20 minutes at 4 °C. After 15 min, 1 μg of anti-KLF12/anti-EP300 suspended in lysis buffer was added to the supernatant, which was collected in an EP tube. Then, this EP tube was incubated on a sky wheel for 1 hour. After 1 hour, the reaction mixtures were incubated with 25 μL of protein A Dynabeads (Invitrogen) at 4 °C overnight. The products of immunoprecipitation were washed six times with IP lysis buffer. The products were added in loading buffer and put in metal bath at 100 °C for 15 minutes. Then, the beads were washed and recovered using magnets.

**RNA-FISH for LncRNA-PACERR**

It was as previously described for RNA-FISH (23). Diethyl pyrocarbonate (DEPC) was used to fix THP-1 for more than 12 hours. Next, THP-1 were dehydrated by an alcohol gradient and embedded in paraffin. Fluorescence-labelled single-strand probes were hybridized. PACERR oligos were gotten from Servicebio Technology. After labelling, images were taken with a fluorescence microscope (Zeiss).

The probe sequence of LncRNA-PACERR is TCTTCTGTCCCGACGTGACTTCCTCGACCCTCTA.

The probe sequence of 18s RNA is ATGCTTTCGCTCTGGTCCGTCTTGC.

The probe sequence of U6 is CACGAATTTGCGTGTCATCCTT.

**Immunofluorescence (IF)**

PDAC tissues were fixed with 4% paraformaldehyde (PFA) for 30 minutes and embedded in OCT. The appropriate primary and secondary antibodies were used to perform immunostaining. Nuclei was counterstained with DAPI respectively. Images were taken with a SP-8 con-focal Microscope.

**Immunohistochemistry staining on PDAC tissue microarrays**

Tissue microarrays (TMAs) containing paraffin sections from 110 PDAC patients (2016 year to 2017 year) from Ruijin Hospital were immunostained for CD163, CD206, CD80, KLF12 and Ki-67 proteins. TMAs were fixed in 40% paraformaldehyde overnight at 4°C and dehydrated by an alcohol gradient. Anti-CD163, anti-CD206, anti-80 and anti-KLF12 were used as primary antibodies.

**Cell proliferation assay**

The CCK-8 (Cell count kit-8) assay (Meilunbio, Dalian, China) was used to determine cell proliferation. Transfected Pancreatic cancer cells (2 × 10^3^) were collected at 48 h post-transfection and plated in a 96-well plate and supernatant from THP-1 was used to incubate for another 5 days. Then, 10 µL of CCK-8 assay reagent was added at specific times and incubated for another 2 h, followed by measuring the absorbance at 450 nm. For the colony formation assay, 2 × 10^3^ cells/well were plated in 6-well plates and co-cultured with THP-1 cells and used to assess the proliferative ability of pancreatic cancer cells. After 2 weeks, the cells were fixed using 1 % crystal violet stain solution for 20 min at room temperature, and the number of colonies was counted manually.

**Transwell invasion and migration assay**

5 × 10^4^ PANC-1/PATU-8988 WT cells were seeded to the upper chamber in 200 μL DMEM without serum, 3× 10^4^THP-1 cells were seeded to the lower chamber (Corning Life Sciences, Corning, NY, USA) in 400 μL RPMI-1640 with 20% FBS, and the chambers were put into 37 °C constant temperature incubators for one day for migration assay. For the transwell invasion assay, the upper chamber was coated with 1.25 mg/ml of Matrigel (50μL/well) (BD, USA) and cultured for 2 days. Afterwards, the migrated or invaded cells were stained with crystal violet and counted in 3 random fields.
