# Supplementary material for: LncRNA-PACERR induces pro-tumour macrophages via interacting with miR-671-3p and m6A-reader IGF2BP2 in pancreatic ductal adenocarcinoma
Source: J Hematol Oncol. 2022 May 7;15:52. doi: 10.1186/s13045-022-01272-w (PMC9077921; doi:10.1186/s13045-022-01272-w)
Supplement: Supplementary file 8 — Additional file 8. Supplementary Figure legends. [file 13045_2022_1272_MOESM8_ESM.docx]

**Supplementary Table legends and Figure legends**

**Supplementary Table 1** Clinicopathologic characteristics of PDAC patients from Ruijin Hospital in a cDNA microarray of Macrophages.

**Supplementary Table 2** Clinicopathologic characteristics of PDAC patients from Ruijin Hospital in a tissue array.

**Supplementary Table 3** Sequences targeting Related Genes.

**Supplementary Table 4** All primer sequences used in the qPCR process.

**Supplementary Table 5** Antibodies used for western blotting/flow cytometry/MACS/RIP/ChIP/co-IP.

**Supplementary Figure 1** The prognosis of patients with high CD163+ TAMs infiltration was better than those with low infiltration.

(A) Representative images of IHC (CD163 and CD80) of tumour tissue and normal tissue from PDAC patients.

(B) Kaplan-Meier survival curve presenting the overall survival of 110 PDAC patients, grouped according to the extent of CD163^+^ TAM infiltration.

**Supplementary Figure 2** Knockdown of LncRNA-PACERR hinders pro-tumour functions of THP-1-derived TAMs *in vitro.*

(A) qPCR analysis of LncRNA-PACERR expression after LncRNA-PACERR knocked down in THP-1 derived TAMs.

(B) and (C) THP-1 derived TAMs or PATU-8988/PANC-1 cells (co-cultured with PATU-8988 (B) or PANC-1 (C)) were subjected to CCK-8 assays. ^*^P＜0.05; ^**^P＜0.01; ^***^P＜0.001; ^****^P＜0.0001.

(D) Proliferation capacity of PATU-8988 or PANC-1 cells co-cultured with THP-1-derived TAMs or PATU-8988/PANC-1 cells.

(E) and (F) THP-1 derived TAMs (co-cultured with PATU-8988 (E) or PANC-1 (F)) were subjected to CCK-8 assays after transfection with sh-LncRNA-PACERR.

**Supplementary Figure 3** LncRNA-PACERR^+^ TAMs facilitate primary PDAC cell (0037) growth and liver metastasis in vivo.

(A) Representative images of IHC (Ki-67) and Ki-67 H-score of subcutaneous tumour tissues from groups of THP-1 shNC+PATU-8988 and THP-1 sh1 PACERR+PATU-8988. ^*^P＜0.05; ^**^P＜0.01; ^***^P＜0.001; ^****^P＜0.0001.

(B) Images of BALB/c nude mice which were co-injected with THP-1 cells and 0037 cells subcutaneously. (n=6 per group)

(C) Images of subcutaneous tumours. (n=6 per group)

(D) and (E) The tumour weights and volumes of the subcutaneous xenografts.

(F) Representative images of liver metastasis and the number of metastatic cells in PDAC mouse model, in which 0037 cells mixed with TAMs (THP-1 shNC/sh1 LncRNA-PACERR) were injected into the spleens of BALB/c nude mice. Data is shown as the results from three independent experiments. ^*^P＜0.05; ^**^P＜0.01; ^***^P＜0.001; ^****^P＜0.0001.

**Supplementary Figure 4** miR-671-3p is involved in the effects of LncRNA-PACERR to promote the M2 polarization in PDAC.

(A) Fluorescence in situ hybridization (FISH) of 18sRNA/U6 (red) in THP-1-derived TAMs (co-cultured with PATU-8988 or PANC-1). DAPI staining (blue) shows the nuclei. Scar bar: 20μm.

(B) and (D) qPCR analysis of miR-671-3p expression in THP-1 derived TAMs (NC/miR-671-3p mimics /shLncRNA-PACERR/shLncRNA-PACERR+miR-671-3p inhibitor)

(C) RIP qRT-PCR detecting the enrichment of Biotin-labeled LncRNA-PACERR in the miR-671-3p in LncRNA-PACERR KD THP-1 derived TAMs after RNA pull-down assay (n = 3). ^*^P＜0.05; ^**^P＜0.01; ^***^P＜0.001; ^****^P＜0.0001.

(D) qPCR analysis of the relative expression of M2 markers (Arginase-1, CD163, TGFβ, CD206, IL-10 and IL-6) and M1 marker (CD80, IL-1β) in THP-1-derived TAMs (Crtl/shLncRNA-PACERR/shLncRNA-PACERR + miR-671-3p inhibitor). Data is shown as the results from three independent experiments.

(E) Flow cytometry analysis of the expression of M2 markers (CD163 and CD206) in THP-1-derived TAMs (Crtl/shLncRNA-PACERR/shLncRNA-PACERR + miR-671-3p inhibitor). Data is shown as the results from two independent experiments. ^*^P＜0.05; ^**^P＜0.01; ^***^P＜0.001; ^****^P＜0.0001.

**Supplementary Figure 5** miR-671-3p is involved in the effects of LncRNA-PACERR to facilitate pro-tumour functions in PDAC.

(A) and (B) THP-1 derived TAMs (co-cultured with PATU-8988 (A) or PANC-1 (B)) were subjected to CCK-8 assays after transfection with sh-LncRNA-PACERR and miR-671-3p inhibitor.

(C)-(E) Proliferation (C), migration (D) and invasion (E) capacity of PATU-8988 or PANC-1 cells co-cultured with THP-1-derived TAMs (Crtl/shLncRNA-PACERR/shLncRNA-PACERR + miR-671-3p inhibitor). Crtl means that cells were transfected in negative control plasmids. ^*^P＜0.05; ^**^P＜0.01; ^***^P＜0.001; ^****^P＜0.0001.

**Supplementary Figure 6** miR-671-3p+ TAMs facilitate pro-tumour functions in PDAC.

(A)-(C) Proliferation (A), migration (B) and invasion (C) capacity of PATU-8988 or PANC-1 cells co-cultured with THP-1-derived TAMs (Crtl/miR-671-3p mimics/miR-671-3p inhibitors). Crtl means that cells were transfected in negative control plasmids. ^*^P＜0.05; ^**^P＜0.01; ^***^P＜0.001; ^****^P＜0.0001.

**Supplementary Figure 7** KLF12 is involved in the effects of LncRNA-PACERR to promote the M2 polarization in PDAC.

(A) The protein expression of NMT-1 in THP-1 derived TAMs (co-cultured with PANC-1 or PATU-8988 cells).

(B) Colocalization of CD206 (green) and KLF12 (red) in 110 clinical samples of pancreatic ductal adenocarcinoma (PDAC) as shown by fluorescence microscopy. DAPI staining (blue) shows the nuclei (DNA). Scar bar: 50μm.

(C) Kaplan-Meier survival curve presenting the overall survival of 110 PDAC patients, grouped according to the extent of KLF12^+^ TAM infiltration.

(D) co-localization of LncRNA-PACERR and KLF12 in THP-1 derived TAMs by using FISH and IF assay.

(E) The protein expression of KLF12, c-myc and IGF2BP2 in TAMs which were isolated from subcutaneous tumours (THP-1+PATU-8988) by MACS.

(F) qPCR analysis of the relative expression of M2 markers (Arginase-1, CD163, TGFβ, CD206, IL-10 and IL-6) and M1 marker (CD80, IL-1β) in THP-1-derived TAMs (Crtl/shLncRNA-PACERR/shLncRNA-PACERR + KLF12 OE). Data is shown as the results from three independent experiments.

(G) Flow cytometry analysis of the expression of M2 markers (CD163 and CD206) in THP-1-derived TAMs (Crtl/shLncRNA-PACERR/shLncRNA-PACERR + KLF12 OE). Data is shown as the results from two independent experiments. ^*^P＜0.05; ^**^P＜0.01; ^***^P＜0.001; ^****^P＜0.0001.

**Supplementary Figure 8** KLF12 is involved in the effects of LncRNA-PACERR to facilitate pro-tumour functions in PDAC.

(A) and (B) THP-1 derived TAMs (co-cultured with PATU-8988 (A) or PANC-1 (B)) were subjected to CCK-8 assays after transfection with sh-LncRNA-PACERR and KLF12 OE.

(C)-(E) Proliferation (C), migration (D) and invasion (E) capacity of PATU-8988 or PANC-1 cells co-cultured with THP-1-derived TAMs (Crtl/shLncRNA-PACERR/shLncRNA-PACERR + KLF12 OE). Crtl means that cells were transfected in negative control plasmids. ^*^P＜0.05; ^**^P＜0.01; ^***^P＜0.001; ^****^P＜0.0001.

**Supplementary Figure 9** KLF12^+^ TAMs facilitate pro-tumour functions in PDAC.

(A)-(C) Proliferation (A), migration (B) and invasion (C) capacity of PATU-8988 or PANC-1 cells co-cultured with THP-1-derived TAMs (Crtl/shKLF12/KLF12 OE). Crtl means that cells were transfected in negative control plasmids. ^*^P＜0.05; ^**^P＜0.01; ^***^P＜0.001; ^****^P＜0.0001.

**Supplementary Figure 10** KLF12 binds directly to LncRNA-PACERR in a LncRNA-PACERR-dependent manner.

(A) The motif of KLF12 in Homo sapiens.

(B) Association of H3K27me3 with the promoter region of LncRNA-PACERR in THP-1-derived TAMs (Crtl/KLF12 OE / shKLF12) analyzed by ChIP-qPCR.

(C) and (D) The prediction binding sites between LncRNA-PACERR and KLF12 by carRAID.

(E) THP-1 cells were infected with negative control-Flag lentivirus or lentiviral virus encoding Flag-tagged KLF12 transcripts with or without the predicted RNA binding region (KLF12-Flag or KLF12-Mutant-Flag) and stimulated into TAM models before RIP assays. Whole-cell lysates were subjected to immunoprecipitation with the indicated antibodies. Eluted RNAs were reverse transcribed, and qPCR was performed with primers specific for PACERR. Normal rabbit IgG was used as a negative control.

**Supplementary Figure 11** LncRNA-PACERR interacts with m6A reader IGF2BP2.

(A) Silver staining of LncRNA-PACERR-associated proteins.

(B) The sequence of the IGF2BP2 peptide after mass spectrometry.

**Supplementary Figure 12** LncRNA-PACERR and IGF2BP2 regulate KLF12 and c-myc.

(A) The prediction binding sites between LncRNA-PACERR and IGF2BP2 by carRAID.

(B) Immunoblot of the FLAG for LncRNA-PACERR enrichment in HEK293T cells transfected with the FLAG-tagged full-length or truncated IGF2BP2 constructs (n = 3). aa, amino acid.

(C) The qRT-PCR analysis (top) of LncRNA-PACERR and immunoblot (down) of IGF2BP2 in IGF2BP2 OE THP-1 derived TAMs (n = 3).

(D) The qRT-PCR analysis (top) and immunoblot (down) of IGF2BP2 in LncRNA-PACERR OE THP-1 derived TAMs (n = 3).

(E) and (F) The qRT-PCR analysis of LncRNA-PACERR in IGF2BP2 KD THP-1 derived TAMs with ectopically expressed LncRNA-PACERR (E) and in LncRNA-PACERR KD THP-1 derived TAMs with ectopically expressed IGF2BP2 (F) (n = 3).

(G) and (H) The qRT-PCR analysis of KLF12 in IGF2BP2 KD THP-1 derived TAMs with ectopically expressed LncRNA-PACERR (G) and in LncRNA-PACERR KD THP-1 derived TAMs with ectopically expressed IGF2BP2 (H) (n = 3).

(I) and (J) The qRT-PCR analysis of c-myc in IGF2BP2 KD THP-1 derived TAMs with ectopically expressed LncRNA-PACERR (I) and in LncRNA-PACERR KD THP-1 derived TAMs with ectopically expressed IGF2BP2 (J) (n = 3).

(K) and (L) Immunoblot of KLF12 and c-myc in LncRNA-PACERR KD and IGF2BP2 KD cells (K), and LncRNA-PACERR and IGF2BP2 OE cells (L).

**Supplementary Figure 13** LncRNA-PACERR binds to their targets in an IGF2BP2-dependent manner.

(A) Western Blot analysis of the expression of Mettl3, Mettl14 and WTAP in THP-1 derived TAMs.

(B) RIP qRT-PCR detecting the enrichment of IGF2BP2 in the KLF12 5′ UTR in LncRNA-PACERR KD THP-1 derived TAMs (n = 3).

(C) RIP qRT-PCR detecting the enrichment of biotin-labelled LncRNA-PACERR to KLF12 3' UTR in RNA pull-down beads treated with protease K.

(D) and (E) RIP qRT-PCR detection of the enrichment of IGF2BP2 (C) and m6A (D) in the KLF12 3′ UTR WT and MUT luciferase reporters in the LncRNA-PACERR and IGF2BP2 KD cells (n = 3).

(F) Relative luciferase activity levels of KLF12 3′ UTR WT and MUT reporters in the LncRNA-PACERR and IGF2BP2 KD cells (n = 3). ^*^P＜0.05; ^**^P＜0.01; ^***^P＜0.001; ^****^P＜0.0001. “ns” means no statistically significance.
